# Microbial synthesis and extraction of value-added metabolites by *Rhodotorula toruloides* from waste stream: a sustainable approach

**DOI:** 10.1186/s12934-025-02752-7

**Published:** 2025-06-16

**Authors:** L. A. Swagatika Priyadarshini, Rashmi Kataria

**Affiliations:** School of Biosciences and Technology (SBST), Vellore Institute of Technology, Vellore, Tamil Nadu India

**Keywords:** Carotenoids, Extraction methods, Genetic engineering, Lipids metabolic pathways and sustainable carbon sources

## Abstract

*Rhodotorula toruloides* (*R. toruloides*) has shown great potential for the microbiological synthesis of useful substances, including lipids and carotenoids. Through interconnected metabolic pathways, this oleaginous yeast can synthesize various carotenoids and accumulate significant amounts of lipids. The mevalonate pathway plays a crucial role in the production of these substances. *R. toruloides* can utilize diverse carbon sources, including waste-derived and sustainable substrates. The product yields are significantly influenced by the optimization of stress factors and culture conditions. Lipid and carotenoid extraction methods have advanced from traditional approaches to more sophisticated techniques, such as enzyme-assisted extraction, ultrasound-assisted extraction, and supercritical fluid extraction. These modern techniques aim to minimize environmental impact while maximizing efficiency and selectivity. Genetic engineering has played a pivotal role in enhancing lipid and carotenoid accumulation in *R. toruloides*. These strategies involve overexpressing key biosynthetic genes, modifying regulatory elements, and introducing heterologous pathways. Such approaches have expanded the range of chemical synthesis and led to significant improvements in product yields. This study provides a comprehensive insight into the metabolic linkages between lipid and carotenoid biosynthesis, highlighting how stress factors, genetic engineering, and waste-derived substrates influence productivity. Furthermore, present review uniquely explores the role of *R. toruloides* in environmental bioremediation and wastewater treatment, emphasizing its potential for sustainable waste valorization. Due to its ability to synthesize valuable chemicals from a wide array of carbon sources, *R. toruloides* is a promising candidate for commercial applications in the feed, food, cosmetic, and biofuel industries.

## Background

Colors have a big impact on our visual experience. They are frequently the first thing that grabs our attention whenever we come upon something, whether it be food, plants, clothing, animals, or even other people. Due to the tremendous effects of color on the human senses, a wide range of companies have made significant investments in R&D projects aimed to examine new sources and formulations of color [1]. Synthetic dyes, plant-derived pigments, and microbial pigments are the three main sources of color additives used in food, medicine, and cosmetic products. Colorants and pigments are the terms used in the chemical community to describe soluble and insoluble colored substances [2]. All colored compounds, regardless of their solubility, are referred to as pigments in biological terms. The term “bio-pigment” is often used to refer of naturally derived pigments which are different from synthetic pigments that are manufactured in industries [3]. The major sources of natural colors are microbes and plants, each has certain benefits and drawbacks of its own. Plant-based pigments are natural but can become unstable with heat, light, or high pH, causing degradation or structural changes that can result in decreased color intensity and product shelf life. For example, anthocyanins and betalains, commonly found in vegetables and fruits, are sensitive to high temperatures and UV light, resulting in color fading during storage or processing. Furthermore, the availability and consistency of plant pigment supplies in industrial applications are challenging due to their seasonal and geographical accessibility [1, 4, 5]. Plant-based pigments are not soluble in water and are only available in certain seasons or regions which makes it difficult for an adequate supply throughout the year [6]. Triacylglycerols and fatty acids are obtained from plant and animal sources which are important raw materials for oleochemicals and biofuels synthesis. Microbial lipid synthesis has several benefits over extraction from flora and oil crops. These advantages include shorter production cycles, tailored procedures, and improved accessibility to structural diversity. A great effort has been undertaken to design efficient strains for the microbial production of chemicals and biofuels by using lipids as nutrients [7].

## Rhodotorula toruloides

*Rhodotorula toruloides (R*. *toruloides)* (formerly known as *Rhodosporidium toruloides*) is a red-colored, strictly aerobic and heterothallic yeast and was originally isolated from plants wood [8]. *R. toruloides* synthesizes a variety of metabolic products including lipids, enzymes, carotenoids and terpenoids when nutrients including nitrogen, phosphorus are low [9]. The colored carotenoids pigment displays various medicinal and industrial properties and hence, it can be used as a precursor to vitamin A, colorants, and antioxidants [9]. This oleaginous yeast also produces special enzymes which are used in pharmaceuticals and chemicals [10, 11]. Lipids synthesized by *R. toruloides* can be used as an alternative for chemicals and fuel. *R. toruloides* isolates rank second among the high yielding producers of lipids as reported in the literature [12–15]. This microorganism has various biotechnological applications because of its ability to tolerate and use a wide variety of carbon sources, such as lignocellulosic hydrolysates and industrial waste streams. This yeast is more feasible for large-scale bioconversion processes because of its high tolerance to inhibitors like acetic acid and furfural that are frequently present in biomass hydrolysates [16]. However, genetic engineering in *R. toruloides* is challenging due to its thick cell wall, which hinders DNA delivery, and the limited efficiency of homologous recombination for targeted genome editing. Despite of challenging genetic modification efficiencies, several methods are being explored to obtain the high yielding strain. These challenges have been gradually mitigated by advancements in transformation protocols, CRISPR-Cas9 systems, and optimized vectors for metabolic engineering [16]. In industrial bioprocess development a variety nitrogen and carbon sources are being explored to obtain high microbial cell densities for metabolites production [17–19]. Due to its capability to co-ferment xylose and glucose, it is a good candidate for next-generation biorefineries, which would allow for the effective biomass utilization [20, 21].

## Carotenoids and their classification in *R. toruloides*

Carotenoids are 40-carbon isoprenoids found in many fruits and vegetables. These lipid-soluble compounds have antioxidant and immunological properties, protect against eye disorders, LDL (Low-Density Lipoprotein) oxidation, and oxidative stress. [22–25]. Carotenoids are not synthesized in humans and need to be taken as supplement through food [4, 27, 28]. The carotenoids market is expected to rise from its estimated USD 1.57 billion in 2022 to USD 2.09 billion by 2027 with 4.5% compound annual growth rate [26].

β-carotene: **(**C_40_H_56_)**-** It is a fat-soluble carotenoid, composed of two retinyl groups and eleven conjugated double bonds. In addition to being a natural colorant, β-carotene is a dietary supplement which acts as a provitamin and antioxidant agent [29, 30]. It is extensively utilized in nutrition, medicine, food additives, cosmetics [31], and supplements for animal feed [32]. According to research, beta-carotene also has potential health benefits, including the prevention of tumors, malignancies, and heart problems [30, 33–36].

Torulene: Torulene (C_40_H_54_) is an important carotenoid compound commonly found in red yeast that comes under *Rhodotorula* genus and is primarily generated in yeast cells through the mevalonate pathway [37, 38]. It contains 13 conjugated double bonds and a single β-ionone cyclic ring and poses better antioxidant qualities than β-carotene [39, 40]. The genera *Sporidiobolus, Rhodotorula, and Sporobolomyces* are potential sources of torulene producing yeasts [38]. A large-scale production requires optimized processes and careful strain selection. These antioxidant-rich compounds show great potential as safe additives in dietary supplements, cosmetics, and pharmaceuticals [23, 38, 41].

Torularhodin: Torularhodin (C_40_H_52_O_2_) is a carotenoid compound similar to torulene but with an additional oxygen-containing functional group. Similar to torulene, torularhodin is also produced via the mevalonate pathway in yeast cells, particularly in species such as *Rhodotorula *[37, 38]. It shares some of the antioxidant properties of other carotenoids, offering protective effects against oxidative damage [39, 40]. Due to its antioxidant and potential bioactive properties, torularhodin is being explored for use in cosmetic formulations, dietary supplements, and possibly in pharmaceutical applications [23, 38].

## Lipids and their classification in *R. toruloides*

Lipids are essential, high-energy molecules that serve as key energy storage units for cellular processes and are fundamental structural components of cellular membranes. Lipids have an important role in the basic functions of life by regulating physiological processes and help in maintaining cellular homeostasis [42]. *R. toruloides* can accumulate over 20% of its biomass as lipids, making it a suitable option for microbial oil production [42, 43]. Fatty acid biosynthesis in *R. toruloides* follows a common pathway where acetyl-CoA is converted to malonyl-CoA, which then undergoes multiple cycles of elongation, reduction, and dehydration to form long-chain fatty acids. These processes are catalyzed by the fatty acid synthase (FAS) complex and are influenced by precursor availability, enzyme activity, and metabolic regulation. C16:1 (palmitoleic acid), C18:0 (stearic acid), C16:0 (palmitic acid), C18:2 (linoleic acid) and C18:1 (oleic acid) are the most common fatty acids produced by *R. toruloides *[44, 45]. Microbial lipids can increase the bioavailability of carotenoids by serving as lipid carriers. These lipid molecules improve the solubility of carotenoids by formation of micelles. Additionally, lipids help to integrate carotenoids into biological membranes and enhance their antioxidant properties [4, 4, 46]. Addition to this, fatty acids can be utilized as raw materials for the production of biofuels and bio-based chemicals [45, 47, 48].

Palmitic acid: Enzymatic processes in the fatty acid biosynthesis pathway produce palmitic acid, a crucial saturated fatty acid in *R. toruloides*. In order to generate a 16-carbon chain, acetyl-CoA must be converted to malonyl-CoA. This is followed by many cycles of condensation, reduction, and dehydration [49].

Palmitoleic acid: *R. toruloides* uses its lipid biosynthesis pathways to produce palmitoleic acid, which is a monounsaturated fatty acid with a single double bond in its hydrocarbon chain. This essential fatty acid may help to reduce a risk of cardiovascular disease, inflammation and enhances overall metabolic health [49].

Stearic acid: *R. toruloides* produces stearic acid (CH_3_(CH_2_)_16_COOH), It is an 18-carbon saturated fatty acid as a byproduct of lipid production [50]. The biosynthesis of this compound in *R. toruloides* is dependent on the availability of precursors, activity of the fatty acid synthase (FAS) complex, and the presence of fatty acid modification enzymes [28].

Oleic acid: Oleic acid (C18:1) is a monounsaturated fatty acid produced in *R. toruloides* through the desaturation of stearic acid (C18:0) [51]. Malonyl-CoA, which is an intermediate metabolite is enzymatically converted to stearic acid by fatty acid synthase complex (FAS) [52]. Stearoyl-CoA desaturase (SCD) introduces a double bond at the ninth carbon position of stearic acid, converting it into oleic acid. Oleic acid plays a crucial role in membrane fluidity, lipid storage, and serves as a precursor for polyunsaturated fatty acids like linoleic acid (Fig. 2) [33].

Linoleic acid: It is produced in *R. toruloides* by oleic acid desaturase which adds a double bond at the 12 th carbon that results in desaturating oleic acid (C18:1) [51]. Linoleic acid is an essential fatty acid needed for processes like signaling, membrane fluidity, and lipid synthesis. Since the body cannot produce it, people must obtain it from food or supplements [52].

## Mechanism of carotenoid biosynthesis pathway in *R. toruloides*

**Fig. 1:**
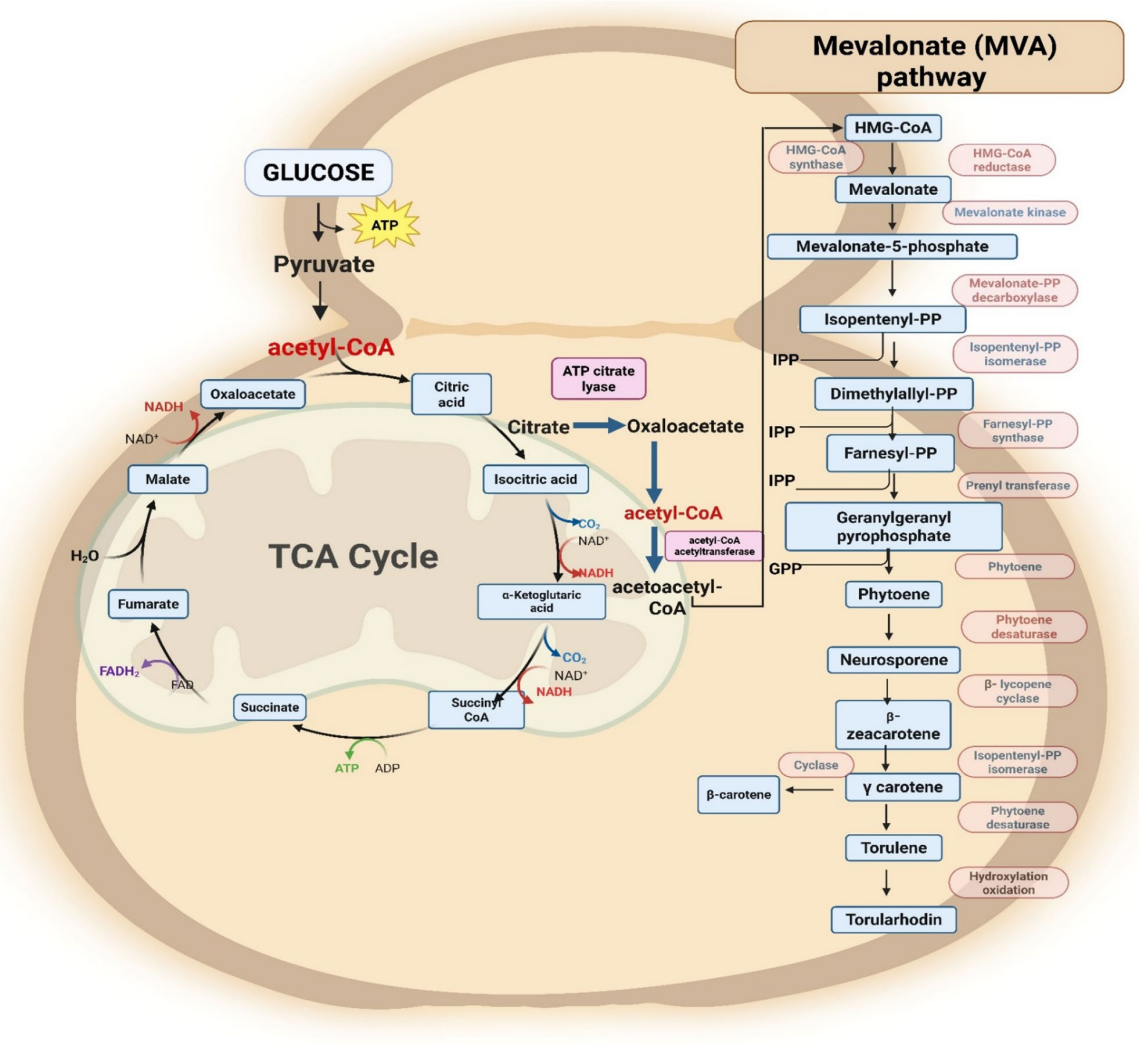
Schematic representation of the TCA cycle and the Mevalonate (MVA) pathway leading to carotenoid biosynthesis. Glucose-derived pyruvate enters the TCA cycle that generates intermediates and acetyl-CoA, which feeds into the MVA pathway for the bio synthesis of carotenoids in *R. toruloides* (substrates involved: HMG-CoA-3-hydroxy-3-methylglutaryl-CoA, IPP-Isopentenyl diphosphate, GPP-Geranyl diphosphate) (Products: 2 ATP, 8 NADH, 2 FADH2, 6 CO2) (Created with Bio render)

The biosynthesis of carotenoids in *R. toruloides* is a complex, multi-step process that occurs in two major phases. In first phase the carbon source synthesizes isoprene precursors and in second phase these precursors are enzymatically converted into various carotenoids [53, 54]. The process begins with the uptake of carbon sources from the culture medium, which are metabolized into intermediate molecules that enter the mevalonate (MVA) pathway. This pathway starts with acetyl-CoA as the primary substrate, which undergoes condensation catalyzed by acetyl-CoA acetyltransferase 2 (ACAT2) to form acetoacetyl-CoA (Fig. 1). The enzyme 3-hydroxy-3-methylglutaryl-CoA synthase (HMGS) then converts acetoacetyl-CoA into 3-hydroxy-3-methylglutaryl-CoA (HMG-CoA). Following this, 3-hydroxy-3-methylglutaryl-CoA reductase (HMGR) catalyzes the reduction of HMG-CoA to mevalonate (MVA), a crucial intermediate in isoprenoid biosynthesis. Mevalonate undergoes phosphorylation by mevalonate kinase (MK) to form mevalonate 5-phosphate (MVP), which is further phosphorylated by phosphomevalonate kinase (PMK) to yield mevalonate-5-diphosphate (MVPP). The final step in this phase involves mevalonate diphosphate decarboxylase (MDD), which catalyzes the decarboxylation of MVPP, producing isopentenyl pyrophosphate (IPP), the essential C5 building block for carotenoid biosynthesis [55, 56]. In the second phase, IPP undergoes an isomerization reaction catalyzed by isopentenyl pyrophosphate isomerase to form dimethylallyl pyrophosphate (DMAPP). Through sequential enzymatic reactions, three molecules of IPP are added to one molecule of DMAPP, leading to the synthesis of geranyl pyrophosphate (GPP) then converted to farnesyl pyrophosphate (FPP), and finally formation of geranylgeranyl pyrophosphate (GGPP). GGPP is a 20-carbon isoprenoid that serves as the immediate precursor for carotenoid biosynthesis [53, 57]. The condensation of two GGPP molecules, catalyzed by phytoene synthase, results in the formation of phytoene, which is a first carotenoid in the pathway. Phytoene undergoes a series of desaturation reactions catalyzed by phytoene desaturase (CAR1), forming neurosporene, and ultimately lycopene, which serves as a crucial branching point in the carotenoid pathway [58, 59]. Lycopene can either be cyclized by lycopene cyclase (CAR2) to form γ-carotene, which contains a single β-ionone ring, or undergo further modifications to produce β-carotene, which has two β-ionone rings. Alternatively, lycopene can be dehydrogenated to form torulene, a carotenoid containing thirteen conjugated double bonds [60, 61]. The final stage of the pathway involves the oxidation of torulene by specific enzymes, resulting in the formation of torularhodin, which contains oxygen functional groups (Fig. 1). This sequential enzymatic conversion demonstrates the complexity of carotenoid biosynthesis in *R. toruloides* and provides valuable insights into its metabolic regulation, which can be leveraged for enhanced carotenoid production in biotechnological applications [40, 60, 62].

## Mechanism of lipid biosynthesis pathway in *R. toruloides*

**Fig. 2:**
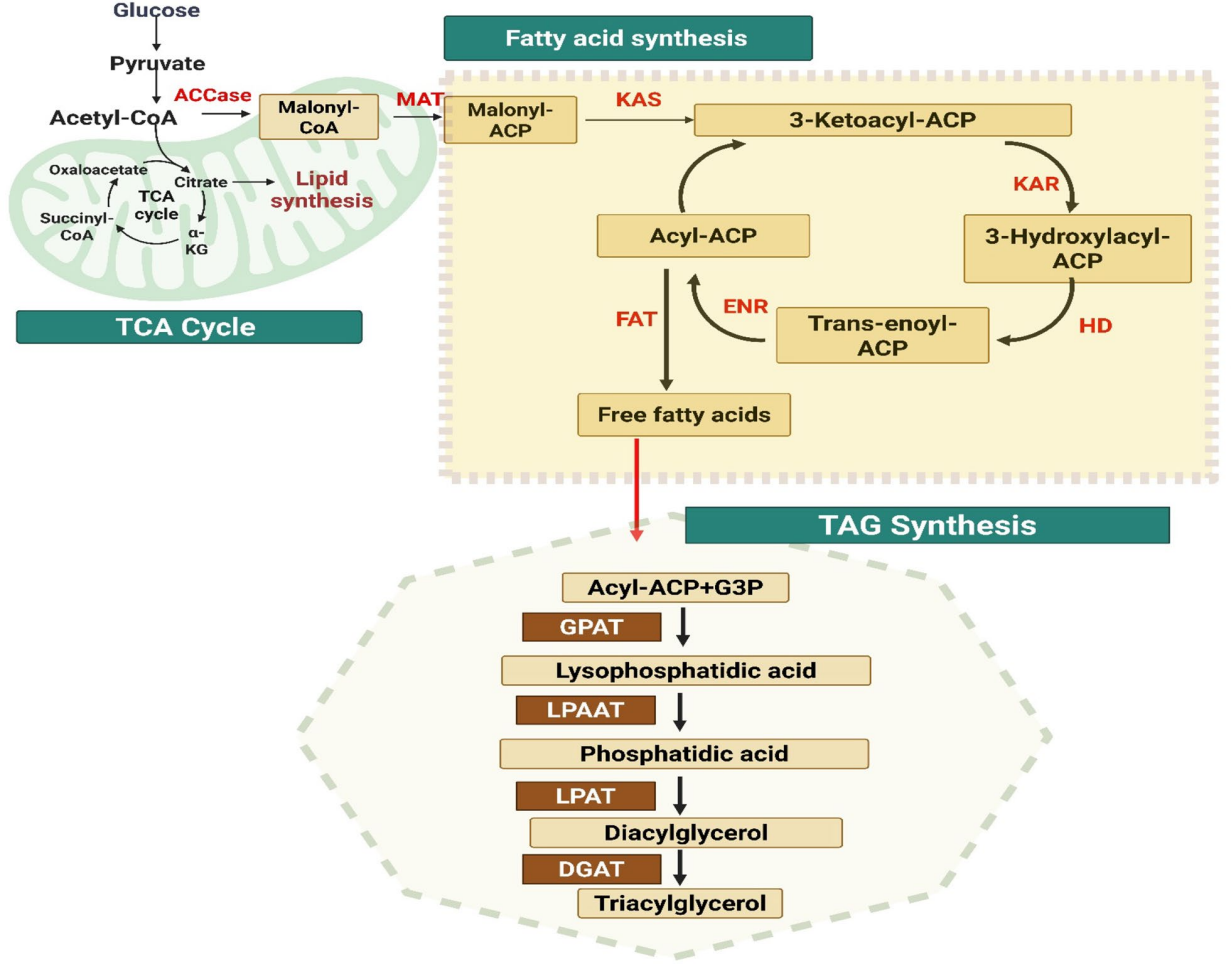
Biosynthesis of lipids—schematic representation of fatty acid and triacylglycerol (TAG) synthesis pathways. Acetyl-CoA derived from the TCA cycle is converted into malonyl-CoA for fatty acid biosynthesis, followed by TAG assembly through a series of enzymatic steps involving GPAT, LPAAT, LPAT, and DGAT in *R. toruloides (*Enzymes involved in various stages of fatty acid and triacylglycerol biosynthesis in *R. toruloides -*ACCase: acetyl-CoA carboxylase, MAT: malonyl-CoA ACP transacylase, KAS: ketoacyl-ACP synthase, KAR: ketoacyl-ACP reductase, HD: hydroxyacyl-ACP dehydratase, ENR: enoyl-ACP reductase, FAT: fatty acyl-ACP thioesterase, GPAT: glycerol-3-phosphate acyltransferase, LPAAT: lysophosphatidic acid, acyltransferase, LPAT: lysophosphatidic acid acyltransferase (same as LPAAT), DGAT: diacylglycerol acyltransferase) (Created with Bio render)

In yeast cells, lipid production occurs in the cytoplasm through a series of enzymatic reaction that converts sugars into long-chain fatty acids which works together with glycolysis, the Krebs cycle, and the citrate/malate shuttle [63]. When nitrogen is low, oleaginous yeasts activate a metabolic pathway to survive. They produce the enzyme AMP deaminase, which converts adenosine monophosphate (AMP) to ammonium ions (NH_4_^+)^ and inosine monophosphate (IMP). This procedure helps the yeast in overcoming restrictions on the availability of external nutrients. The decrease of intracellular AMP levels significantly impacts the tricarboxylic acid cycle (TCA). In oleaginous yeasts, this reduction deactivates isocitrate dehydrogenase (ICDH) which is an enzyme typically activated by allosteric interactions with AMP. The enzyme ICDH, that is found in the mitochondria, is essential for the transformation of isocitrate to α-ketoglutarate [64]. In Fig. 2, acetyl-CoA carboxylase further converts acetyl-CoA into malonyl-CoA. To synthesize acyl-CoA, a fatty acid synthase complex in oleaginous yeast cells needs nicotinamide adenine dinucleotide phosphate (NADPH) as a cofactor in addition to acetyl- and malonyl-CoA. Typically, the pentose phosphate pathway's 6-phosphogluconate dehydrogenase and glucose-6-phosphate dehydrogenase, as well as maleic enzyme (ME), produce NADPH [6]. After their transfer to the endoplasmic reticulum, the resultant acyl-CoA chains undergo the esterification process with glycerol-3-phosphate (G-3-P). Through the Kennedy pathway, this activity produces lipids that are used for storage in the form of TAGs (Triacylglycerols) as well as structural lipids such as glycolipids and phospholipids. Glycerol-3-phosphate acyltransferase (Sct1) acylates glycerol-3-phosphate first in the Kennedy route to produce lysophosphatidic acid. Phosphatidic acid is formed when lysophosphatidic acid is acetylated by lysophosphatidic acid acyltransferase (Slc1) and lysophosphatidylethanolamine acyltransferase (Ale1) [64]. The last steps include phosphatidic acid phosphatase dephosphorylating phosphatidic acid to form diacylglycerol, and diacylglycerol-acyltransferases (Dga1, Dga2) transferring an acyl group from acyl-CoA to the third carbon of diacylglycerol to form TAG [65, 66] (Fig. 2).

## Linkage in carotenoids and fatty acid pathways

**Fig. 3:**
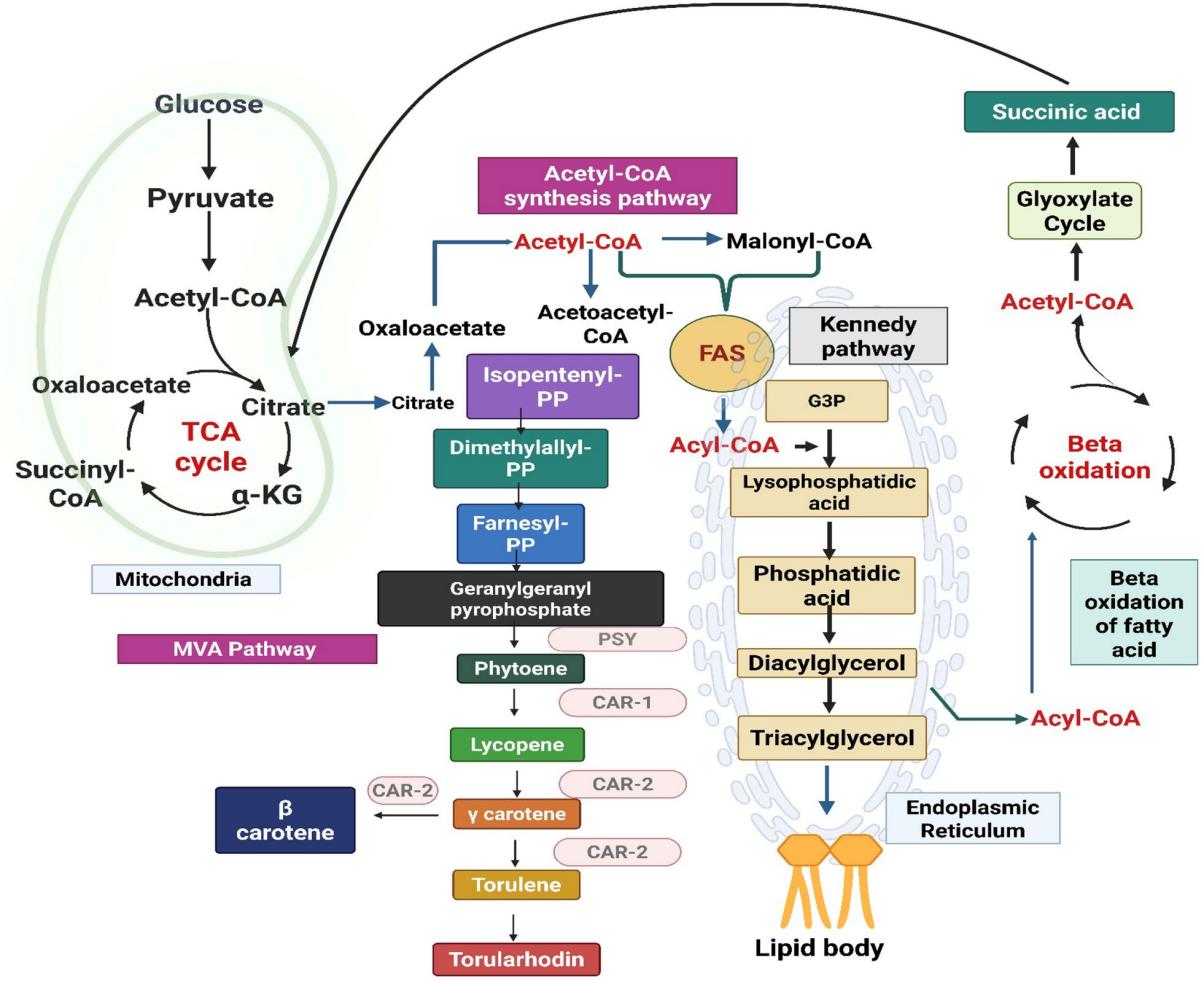
Interconnected metabolic pathways for lipid and carotenoid biosynthesis in *R. toruloides*. The diagram illustrates the TCA cycle, acetyl-CoA synthesis, MVA pathway, Kennedy pathway for lipid formation, and beta-oxidation, showing the metabolic flow toward triacylglycerol and carotenoid production.* (*Enzymes involved: PSY- Phytoene synthase, CAR 1- Phytoene desaturase, CAR 2 -Lycopene cyclase, FAS-Fatty acid synthase, G3P—Glyceraldehyde-3-phosphate *(*Created with Bio render)

*R. toruloides* carotenoid and lipid biosynthesis pathways are linked by isoprenoid precursor molecules, which enable them to exchange intermediates in metabolism and regulatory mechanisms. The carotenoid and lipid biosynthesis pathways depend on the synthesis of isoprenoid precursors such isopentenyl pyrophosphate (IPP) and dimethylallyl pyrophosphate (DMAPP) [67]. The mevalonate pathway begins with acetyl-CoA and involves several enzyme processes to produce these components. Carotenoids, sterols, and lipids are among the various isoprenoid compounds for which the mevalonate-regulated pathway provides essential building blocks [68]. The enzyme, phytoene synthase joins two geranylgeranyl pyrophosphate (GGPP) molecules to start the carotenoid biosynthesis pathway. Phytoene is the product of this chemical process which is the starting point for several carotenoid molecules, including torulene, β-carotene, and lycopene [69]. The broad range of carotenoid molecules are produced by other enzymatic processes that are facilitated by carotenoid desaturases and cyclases. Acetyl-CoA and malonyl-CoA are used in the lipid biosynthesis pathway of *R. toruloides* to create fatty acids, which then combine with glycerol-3-phosphate to form triacylglycerols (TAGs). Precursors for the synthesis of carotenoid and various other lipid components can be obtained via the mevalonate pathway [9]. Isoprenoid precursors (IPP, DMAPP, and GGPP) from the mevalonate pathway connect lipid and carotenoid processes. These precursors link lipid and carotenoid pigment production [61]. The synthesis of lipids and carotenoids in *R. toruloides* can be influenced by the distribution and availability of these precursors. Transcription factors such as *CAR1* (phytoene desaturase) and *CTR9* (Cln Three Requiring 9) regulate the expression of genes involved in the biosynthesis of lipids and carotenoid compounds. Environmental factors like stress and nutrient availability also affect these interrelated metabolic pathways. *CAR1* and *CTR9* positively regulate the expression of genes involved in lipid and carotenoid biosynthesis in *R. toruloides* by influencing the mevalonate pathway to control the production of isoprenoid precursors [68] (Fig. 3).

## Production of lipids and carotenoids in *R. toruloides*

*R. toruloides* is a promising source of carotenoids and lipids, offering advantages over traditional sources. *R. toruloides*-based microbial synthesis is a viable and less expensive alternative in comparison to the extraction from plants. Similarly*, R. toruloides* produces lipids that have potential uses as oleochemicals, nutraceuticals, and feedstocks for biodiesel, mainly in the form of triacylglycerols (TAGs) and free fatty acids (FFAs). *R. toruloides* is a promising host for the synthesis of these important chemicals because it can store up to 70% of its dry cell weight (DCW) as lipids [70].

### Carbon sources

Glucose and sucrose are commonly used as carbon sources for microbial production of carotenoids and lipids. These sugars are the major energy source and the building blocks needed to produce the important metabolites. A monosaccharide, glucose is a widely chosen as the carbon source for a variety of microbes. It is easily broken down by metabolic processes like glycolysis and the pentose phosphate pathway, which generate energy and precursors for lipid and carotenoid pigment accumulation. For the effective synthesis of carotenoids and lipids, glucose is frequently utilized either as the only carbon source or when combined with other sources of carbon [71]. Sucrose is a disaccharide of glucose and fructose which is widely used in industry because it is cheap and easily broken down by microbial enzymes like invertase, leading to high lipid and carotenoid yields [72]. Utilization of these pure sugars for industrial production process is an expensive process. Hence, these sugars can be replaced by sustainable carbon sources such as agriculture and food industry waste streams.

### Renewable carbon sources

The possible alternative of pure sugars are needed to be explored to make production process to be more economical. Different waste streams including lignocellulosic biomass (agricultural leftovers, wood waste, forest waste), industrial by-products (e.g., molasses, whey), and municipal waste streams (e.g., municipal solid waste) are studied widely to be utilised as carbon source. These waste stream-based carbon sources may lower the cost of production [73, 74] (Table 1).

**Table 1:** Production of carotenoids using renewable carbon source and different culture conditions in *R. toruloides*

Micro-organism	Conditions	Carbon source	Pigments and Yield of carotenoids	Nitrogen source	Fermentor study	References
*R. toruloides ATCC 10788 (wild strain)*	Temperature: 30 °C Agitation: 200 rpm pH: 6.0	Tea waste hydrolysate (TWH)	• Torularhodin: 37.6 μg/(DCW), • Torulene: 334 μg/g DCW, β-carotene: 11.96 mg/g DCW	Yeast extract	Shake flask study	[84]
*R. toruloides ATCC 204091 (wild strain)*	Temperature: 25 °C Agitation: 150 rpm pH 5.0	Agricultural market waste	62 mg/L of β-carotene	Yeast extract	Lab scale fermentor study	[117]
*R. toruloides ATCC 10788 (wild strain)*	Temperature: 30 °C Agitation: 200 rpm pH: 6.0	YPG medium	3.6 mg/L of β-carotene	Yeast extract	Shake flask study	[72]
*R. toruloides DSM 4444 (mutant strain)*	Temperature: 30 °C Agitation: 200 rpm pH: 6.0	*Camelina* meal hydrolysate	5.5 mg/L of β-carotene	Yeast extract	Lab scale fermentor	[136]
*R. toruloides ATCC 204091(wild strain)*	Temperature: 25 °C Agitation: 180 rpm pH- 5.5	Rice straw	19 mg/L of β-carotene	Yeast extract	Shake flask study	[70]
*R. toruloides CCT 0783 (wild strain)*	Temperature: 30 °C Agitation: 200 rpm pH: Initial pH of 5.5	Wheat straw	24.58 ± 1.88 mg/L of β-carotene	Yeast extract	Shake flask study	[70]
*R. toruloides ATCC 10788(wild strain)*	Temperature: 30 °C Agitation: 200 rpm pH: Initial pH of 5	Yeast cells, a waste extract	426 μg/g DCW of β-carotene	Ammonium sulfate	Erlenmeyer flasks shaker	[90]
*R. toruloides KP324973(wild strain)*	Batch/fed-batch 11.7 °C, pH 6.1	Corn steep liquor	β-carotene, torulene, torularhodin: Total carotenoid (12.31 mg/g/h)	Yeast extract	Lab scale fermentor study	[119]
*R. toruloides NRRL Y-1091(wild strain)*	Batch Temperature-30 °C, Agitation −250 rpm, 72 h	Wheat straw hydrolysate	14.09 mg/L of β-carotene	–	Lab scale fermentor study	[38]
*R. toruloides NCYC 921(wild strain)*	Fed-batch Temperature-30 °C, Agitation −250 rpm, 72 h	Carob pulp syrup	β-carotene, Torulene, Torularhodin, Total carotenoids (0.42 mg/g)	–	Lab scale fermentor study	[120]
*R. toruloides DSM 4444(mutant strain)*	Batch Temperature- 30 °C, Agitation-160 rpm	*Camelina sativa* meal hydrolysates Flower of *Calendula officinalis*, Zea mays seed flour, potato seed flour	β-Carotene (5.5 mg/L, 12.6 mg/L, 16.0 mg/L)	Yeast extract	Lab scale fermentor study	[82]
*R. toruloides PYCC 5615(mutant strain)*	Batch Temperature: 30 °C, pH 5 Agitation: 250 rpm, 150 h	Sugar beet pulp hydrolysates	5.4 mg/L of β-carotene	Yeast extract	Lab scale fermentor study	[121]

The table outlines strain origin, cultivation parameters, types of carotenoids produced, carbon substrates, carotenoid yields, fermentation scales

**Fig. 4:**
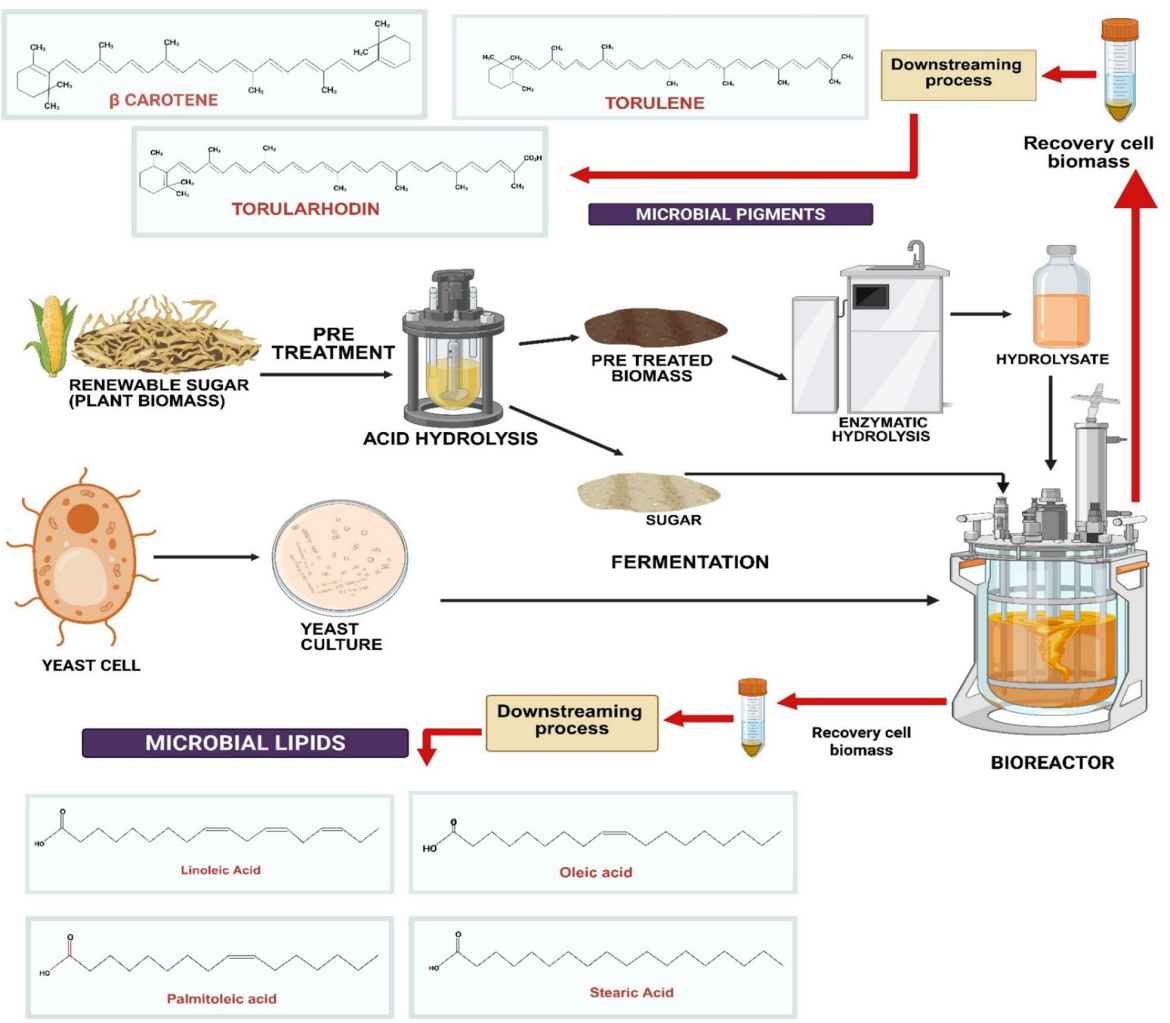
Schematic overview of biomass processing for microbial pigment and lipid production Renewable plant biomass undergoes pretreatment, hydrolysis, and fermentation by yeast cells to produce microbial pigments (β-carotene, torulene, torularhodin) and microbial lipids (linoleic acid, oleic acid, palmitoleic acid, and stearic acid), followed by downstream processing for recovery *(*Created with Bio render)

Lignocellulosic biomass accounts for nearly 50% of the total global biomass with an estimated annual production of 181.5 billion tons, this makes it a low-cost and abundant alternative for industrial microbial fermentation [38]. Its high carbohydrate content, primarily in the form of glucose and xylose makes it a valuable feedstock for microbial growth and lipid production. Lignocellulosic biomass is composed of three primary components: cellulose, hemicellulose, and lignin. Cellulose and hemicellulose serve as fermentable sugar sources while lignin poses challenges due to its complex and recalcitrant structure which can inhibit microbial metabolism [75]. To overcome these challenges, pretreatment and enzymatic hydrolysis are employed to release fermentable sugars such as glucose and xylose, making them more accessible for microbial conversion. Various pretreatment methods are employed to remove lignin from the biomass and further enzymatic hydrolysis process depolymerizes the cellulose and hemicellulose portion to fermentable monomers. The use of lignocellulosic residues including wheat straw [38, 70], rice straw [70], tea-waste hydrolysate [84, 84], agricultural waste [117], *Camelina sativa* meal hydrolysate [136], and sugarcane bagasse offers a potential carbon source. It also reduces dependency on food-based sugars, enhances the economic value of agricultural waste streams, and supports the principles of the circular bioeconomy by promoting the sustainable use of renewable resources. *R. toruloides* is a robust oleaginous yeast and an excellent option for valorizing lignocellulosic hydrolysates due to its ability to metabolize both hexose (e.g., glucose) and pentose (e.g., xylose) sugars [76]. In a study, *R. toruloides* utilized tea waste hydrolysate pretreated with dilute sulfuric acid hydrolysis, yielding 37.6 μg/g DCW torularhodin, 334 μg/g DCW torulene, and 11.96 mg/g DCW β-carotene [84]. *R. toruloides ATCC 10788* and *R. toruloides* NRRL Y-1091, both utilized wheat straw as the carbon source; *R. toruloides* used wheat straw pretreated via ensiling and fungal delignification with *Ceriporiopsis subvermispora*, while *R. toruloides* NRRL Y-1091 used non-detoxified wheat straw hydrolysate, with adaptive inhibitory tolerant strain. It resulted in β-carotene yields of 24.58 ± 1.88 mg/L and 14.09 mg/L, respectively [38, 70, 118, 119]. *R. toruloides* ATCC 204091 (wild strain) utilized agricultural waste as the carbon source after pretreatment and chemical/enzymatic hydrolysis. The β-carotene concentration was observed to be of 62 mg/L [117] (Fig. 4). Furthermore, integrating *R. toruloides* fermentation into modern biorefinery models enables the development of zero-waste production systems, where leftover biomass can be further processed into biofuels or other value-added biochemicals. This approach not only aligns with sustainability and circular bioeconomy principles but also contributes to reducing industrial waste and improving the overall efficiency of bio-based industries [77] (Table 2).

**Table 2:** Lipid production by *R. toruloides* strains under varying culture conditions with different renewable carbon sources

Micro-organism	Origin	Conditions	Lipid	Carbon source	Yield of lipids	Fermentor study	References
*R. toruloides CCT 0783*	Collection Centre Brazil	Temperature: 30 °C Agitation: 200 rpm pH5.0, 6.0, and 7.0	Triacylglycerols (TAGs), Free fatty acids (FFAs)	Glycerol, acetic acid, xylose	20 g/L	Lab scale bioreactors (MiniBio 1000 1.0 L), Fed-batch	[22]
*R. toruloides AS 2.1389*	Obtained from the China MCCC	Temperature:30 °C Agitation: 200 rpm pH: Not mentioned	fatty acid ethyl esters (FAEEs)	Glucose	26 g/L	Flask scale (lab scale cultivation)	[122]
*R. toruloides DMKU3-TK16*	Isolated from soil samples	Temperature: 30 °C Agitation: Not mentioned pH: 6.0	Triacylglycerols (TAGs)	Glucose	9.26 g/L	Lab scale using shake flask cultivation	[90]
*R. toruloides Y4*	cornstalk hydrolysate	Temperature: 30 °C Agitation: 200 rpm pH: 5.5	Triacylglycerols (TAGs), Free fatty acids (FFAs)	Glucose	78.8 g/L	Lab scale using a 15-L bioreactor	[20]
*R. toruloides (strains IFO0880 and IFO0559)*	Obtained from the NBRC culture collection	Temperature: 30 °C Agitation: 200 rpm pH: 6.0	Triacylglycerols (TAGs)	Glucose (70 g/L) and xylose (70 g/L)	16.4 ± 1.1 g/L	Batch shake-flask experiments	[41]
*R. toruloides Y4*	Cornstalk hydrolysate	Temperature: 30 °C Agitation: 200 rpm pH: 6.0	Triacylglycerols (TAGs)	Glucose (70 g/L)	0.21 g/L	Shake-flask cultures of 250 mL Erlenmeyer flasks	[122]
*R. toruloides strains DMKU3-TK16 and NP11*	DMKU3-TK16 (ATCC)	Temperature: 30 °C Agitation:200 rpm pH: 6.0	Triacylglycerols (TAGs)	7% glucose	11.6 g/L	Shake flask cultures	[90]
*R. toruloides Y4*	*R. toruloides* AS 2.1389 obtained from the China General MCCC	Temperature: 30 °C Agitation: 200 rpm pH: 6.0	Triacylglycerols (TAGs)	Glucose	10.3 g/L	Flask scale	[84]
*R. toruloides CBS14*	Obtained from CBS (Centraal bureau voor Schimmelcultures)	Temperature: 30 °C Agitation: 200 rpm pH: 5.5	Triacylglycerols (TAGs)	Glucose, xylose, arabinose	25 g/L	Lab scale in 0.5 L bioreactors	[117]

The table outlines strain origin, cultivation parameters, types of lipids produced, carbon substrates, lipid yields, fermentation scales

## Influence of selected factors in carotenoid and lipid synthesis

### Light

Carotenoids: Several studies have aimed to enhance the yield of certain carotenoids by optimizing the fermentation conditions in *R. toruloides* to increase carotenoid production. Studies indicated the important rise in carotenoid content (up to 70%) at a particular wavelength, especially with blue light [80]. Carotenoid pigments absorb light primarily in the violet to blue-green range of the spectrum. Previous research has shown differences in the ways that different light wavelengths affect the synthesis and composition of carotenoids [78, 79]. Dark conditions were used as a control while cultures were exposed to blue, red, and white light on agar plates. Colonies of *R. toruloides* that were subjected to blue and white light had a deeper red coloration compared with dark-grown and red-light cultures. This study shows that *R. toruloides* is sensitive to blue light [80].

Lipids: It has been demonstrated that *R. toruloides’s* lipid production is enhanced by light exposure and the rise of lipid content (up to 40%) at light conditions of 4000 lx when compared to ideal growth conditions without light. This indicates that metabolic pathways linked to fat accumulation are triggered by light, which is an important factor [81].

### Temperature

Carotenoids: The production of carotenoid pigments and the growth of cells are significantly influenced by temperatures in the environment. In general, microbial cells do not grow or produce high carotenoids yield at cool surroundings (less than 10 °C). On the other hand, high temperatures (more than 25 °C) may inhibit the activity of enzymes required for the synthesis of carotenoids and prevent cells from growing. 10 °C to 25 °C is the ideal temperature range for optimizing the formation of carotenoid pigments as well as biomass accumulation [82]. There is a decrease in carotenoid production at temperatures over 25 °C. Studies show that *R. toruloides*, may enhance carotenoid production at temperatures as low as 15 °C. Carotenoids like β-carotene and torularhodin are produced at a significant rate at these temperatures. Lower temperature-induced stress seems to activate the biosynthetic pathways that produce carotenoids, serving as a defense against oxidative damage [68, 83]*.* Lower temperatures have the ability to both accelerate and slow down the synthesis of carotenoids. This scenario shows how important it becomes to find a balance in terms of optimal conditions for carotenoid production and biomass development [68, 84].

Lipids: When nutrients are limited, especially nitrogen, the oleaginous yeast *R. toruloides* can accumulate almost 70% of its dry weight in lipids. It reaches its maximum lipid synthesis at about 30 °C. Variations in temperature have a significant impact on both the quantity and production of lipids. Some polyunsaturated fatty acids may be produced faster in cool environments, whereas metabolism can speed up during higher temperatures can also cause cell stress that might reduce total lipid yields [85]. *R. toruloides's* fatty acid composition varies with temperature, particularly in terms of its oleic acid concentration. The primary reason may be the denaturation of temperature sensitive enzymes which alters the structure of fatty acids [86].

### Salt concentration

Carotenoids: Osmotic stress and salt concentration are common environmental factors that have a big impact on an organism's development and metabolism. These stresses lead to changes in the amount or composition of cellular carotenoid pigments [87, 88]. In *R. toruloides*, an antioxidant response to hyperosmotic stress, with specific salt concentration boost pigment production. [81]. The addition of 1.0 M sodium and 0.1 M potassium ions raised β-carotene production by 60% [89]. These results highlight the complex interactions between microbial metabolic processes and environmental stresses, suggesting potential strategies to maximize the synthesis of bioactive compounds for commercial use [90].

Lipids: Studies show that *R. toruloides* tolerates up to 6% w/v and lipid formation was significantly enhanced at 4.0% w/v NaCl. In comparison to DCW, this concentration increased lipid synthesis up to 71.3% w/w [91]. The salt affects the metabolic pathways which promote the synthesis of lipids. The results of the study showed that adding NaCl to cultures improves the lipid synthesis and reduces the bacterial contamination that enables more effective growth as well as lipid accumulation in non-aseptic environments [90]. *R. toruloides* is a potential option for biodiesel production. When microbes cultivated in halophilic conditions, a significant increment of oleic acid, palmitic acid, as well as stearic acid was observed [92].

### pH

Carotenoids: The synthesis of lipids and carotenoid pigments is significantly affected by the pH values at which microorganisms grow. Studies on *R. toruloides* show that best carotenoid synthesis occurs at pH 5.0. In order to take advantages of this knowledge, a two-phase pH approach has been developed. The pH is initially set at 4.0 to encourage rapid growth of cells, and it is then changed to 5.0 to optimize the synthesis of carotenoids. When compared to growing at a constant pH, this method significantly increases carotenoid production [81]. It is necessary to balance pigment synthesis and cell growth of *R. toruloides* for industrial scale. An effective way to maximize yields is to use particular pH control techniques including the two-stage approach [93].

Lipids: Studies reveal that *R. toruloides* produces more lipids when the pH is 5.5. When treated with l-proline, the strain reached a maximum cell mass of 14.3 g/L having a lipid content of 58% at this pH, yielding a lipid yield of 0.18 g/g [94].

## Role of genetic engineering in lipid accumulation

Genetically engineered microorganisms can grow more rapidly, also produce higher yields of targeted products and can use a wide range of raw materials (Table 3). The yield of the desired product can be modified by altering gene expression for example, by adding or removing enzymes or by blocking undesirable side reactions. This approach has been used to enhance tolerance to inhibitory compounds and to improve the uptake of both 6-carbon (hexose) and 5-carbon (pentose) sugars. The molecular mechanisms underlying lipid metabolism and its control in the yeast *R. toruloides* are better understood. The integrated information provided by tools like gene sequencing, gene expression pattern analysis, RNA sequencing-assisted annotation, and protein analysis are important for designing genetically engineered microbes [95, 96]. Different genetic modification techniques have been used to increase *R. toruloides'* capacity for lipid synthesis. *Agrobacterium tumefaciens* mediated transformations, lithium acetate-mediated chemical transformations, and the introduction of genetic material through electrical pulses (the electroporation), along with the innovative gene-editing technology CRISPR-Cas9 are important techniques being used. In a shake flask study with synthetic media lipid synthesis was enhanced by a factor of 18.1-fold to 1.95-fold (16 g/L of total lipids) when diacylglycerol acyltransferase (DGA1) and acetyl-CoA carboxylase (ACC1) were overexpressed in *R. toruloides *[97]. Under fed-batch circumstances, *R. toruloides* produces 1.42-fold more lipids when stearoyl-CoA desaturase is overexpressed. This yeast also shows better resistance to furfural and five-hydroxymethyl furfural, which promotes lipid accumulation [85]. Similarly, the fatty acid contents of microbial lipids were altered by overexpressing or silencing the enzymes that regulate fatty acid synthesis [98]. In *R. toruloides,* the elongase (ELO1) gene was overexpressed and knocked down Δ12 Desaturase (FAD2) gene, leading to a 23% rise in the oleic acid quantity. In general, microbial oil's oxidative and thermal stability increases with high oleic acid levels. By inserting the 3-ketoacyl-CoA synthase (KCS) gene into the genome of *R. toruloides* CECT 13085, the strain was genetically modified to produce oils rich in very long-chain fatty acids (VLCFAs) [86]. According to the research, the recombinant *R. toruloides* strain exhibited a 27% relative abundance in the synthesis of VLCFAs, which translated into a yield of 7.9 g/L nervonic acid and 5.8 g/L erucic acid [97] (Table 3).

**Table 3:** Genetic modifications of *R. toruloides* strains to enhance lipid production and metabolite yields from glucose and related substrates

Micro-organism name	Gene studied and target enzyme	Substrate used	Yield	References
*R. toruloides ATCC 10657*	ELO1 (Elongase −1, Delta-12) FAD2 (Fatty acid desaturase)	Glucose	ELO-1 overexpression and FAD-2 deletion cause a 23% rise in oleic content	[86]
*R. toruloides IFO088*	ACC1 (Acetyl-CoA carboxylase), DGA1(Diacylglycerol acyltransferase)	Glucose at a conc. 80 g/L	When both enzymes were overexpressed, 70 g/L of glucose and xylose were used to produce 16.4 g/L and 9.5 g/L of lipid, respectively	[85]
*R. toruloides NP11*	FAD2 (fatty acid desaturase)	Glucose	Linoleic acid is increased fivefold and reaches 1.3 g/L upon overexpression	[37]
*R. toruloides CECT 13085*	KCS (3-ketoacylCoA synthases)	Glucose at a conc. 40 g/L	Microorganism produces long-chain fatty acids (VLCFAs) like erucic acid (5.8 g/L) and nervonic acid (7.9 g/L)	[86]
*R. toruloides CECT 13085*	FAC (Fatty acyl-CoA reductase)	Glucose at a con. 40 g/L	Overexpression of FAC produces ~ 8 g/L of C16-C18 fatty alcohol	[123]
*R. toruloides ATCC 10657*	KU70 DNA end-binding proteins	Glucose conc. 20–80 g/L,	KU70 deficient strains resulted in improved gene deletion frequency	[124]
*R. toruloides IFO0880*	ME (Malic enzyme) SCD (Stearoyl-CoA desaturase)	Glucose, at conc. 80 g/L -batch 600 g/L- fed-batch,	Overexpression increased lipid production to 89.4 g/L	[85]
*R. toruloides IFO0880*	BIS (Bisabolene synthase)	Glucose, xylose, sucrose, lignocellulosic hydrolysates, Corn stover, Switchgrass, Miscanthus hydrolysate	Leads to the production of 680 mg/L of bisabolene	[39]
*R. toruloides NP11*	PTA (Phospho trans-acetylase)	Glucose at a conc. 60 g/L	Comparing to the original strain, modified strain increased lipid production by 64.4% (or 1.20 g/L/d) and cell mass by 8.5% (12.8 g/L) in experiments	[123]

## Role of genetic engineering in carotenoids accumulation

Genetic engineering is used for modifying the yeast’s metabolism to increase the microbial accumulation of carotenoids. In a study, metabolic engineering was used to modify *R. toruloides* to produce more β-carotene by overexpressing endogenous carotenogenic genes, such as lycopene cyclase *(crtYB),* phytoene synthase *(crtYB*), and phytoene desaturase *(crtI) *[55]. The β-carotene concentration of the transformed strain increased by 3.5 times as compared to the wild strain. [39]. The overexpression of *crtYA* boosted carotenoid synthesis, especially β-carotene and elevated the carotenogenic genes expression [55]. To optimize growth conditions for higher carotenoids production in recombinant *R. toruloides* strains, numerous studies have focused on improving cultivation conditions, including carbon sources, nitrogen sources, and environmental parameters. The transcription factors can be manipulated by making genetically engineered strain and activation and repression of key biosynthetic genes. In a study, overexpression of genes including *phytoene synthase* (PSY) or *phytoene desaturase* (PDS) can increase carotenoid production [39, 55].

A study tested different lignocellulosic biomass hydrolysates as substrates for carotenoid production in modified *R. toruloides* strains. [68] (Table 4). Researchers at the Technical University of Denmark genetically engineered *R. toruloides* to produce torularhodin by introducing a heterologous carotenoid biosynthesis pathway. The expression of the yeast gene *crtR* from *Sporobolomyces shibatanus* and the bacteria's *crtYB, crtI, and crtS* genes from *Deinococcus radiodurans* were used in this experiment [55] (Table 4).

**Table 4:** Genetic and mutagenic strategies to enhance carotenoid and pigment production in *R. toruloides* strains

Yeast name	Pigments	wild/mutant strain	Genes studied	Yield	Substrate used	References
*R. toruloides WP1*	β-carotene	Wild	*crtYB, crtI, crtXE*	18.3 mg/g DCW	Olive mill wastewater	[67]
*R. toruloides WB41*	β-carotene	Wild	*crtYB, crtI*	• β-carotene yield: 115.8 μg/g DCW	Glucose	[125]
*R. toruloides ATCC 10657*	Torulene, torularhodin, β-carotene	Mutant	*crtI, crtYB, crtS,crtR*	Wild-type strain: 10.5 mg/g DCW • *crtYB* mutant: 15.3 mg/g DCW • *crtI* mutant: 16.8 mg/g DCW • *crtR* mutant: 14.6 mg/g DCW	Glucose	[124]
*R. toruloides WP1*	Astaxanthin	Mutant	*crtS, crtW, crtZ, crtYB*	14.9 mg/L of total carotenoids mutant strains	Glucose	[111]
*R. toruloides Y4*	Torulene and torularhodin	Wild	ARTP mutagenesis for improved carotenoid production	Max. carotenoid yield of 15.6 mg/L	Tea waste hydrolysate	[84]
*R. toruloides NP11*	Torulene and torularhodin	Wild	Genes involved in xylose metabolism pathway, including *XYL1, XYL2, XKS1*	• xylose—2.2 mg/g DCW • glucose—5.5 mg/g DCW	Glucose, Xylose	[81]
*R. toruloides NP11*	Torulene and torularhodin	Wild	random insertional mutagenesis *RTO4, RHO3, RHO4, and RHO5*	Not specified	Glucose	[60]
*R. toruloides* AS 2.1389	Torulene, torularhodin, and β-carotene	Wild-type	Mutants obtained from random Agrobacterium tumefaciens-mediated transformation	Wild-type: 4.83 mg/g DCW • Mut-1: 9.27 mg/g DCW • Mut-2: 10.86 mg/g DCW • Mut-3: 11.49 mg/g DCW	Glucose	[126]
*R. toruloides AS 2.1389*	Torulene, torularhodin, and β-carotene	Wild-type: *R. toruloides* AS 2.1389, Mutant: *R. toruloides* ΔcrtYB	*crtYB* gene, encoding a cytochrome P450 enzyme	• Wild-type: 4.83 mg/g DCW • *ΔcrtYB* mutant: 7.96 mg/g DCW	Glucose	[69]
*R. toruloides* AS 2.1389	Torulene, torularhodin, and β-carotene	Wild-type: *R. toruloides* AS 2.1389, Mutant: *R. toruloides*Mut-8,Mut-27	*crtI, crtYB, crtS, crtR*	Wild-type: 4.83 mg/g DCW • Mut-8: 9.27 mg/g DCW • Mut-27: 13.39 mg/g DCW	Glucose	[18]
*R. toruloides* AS 2.1389	Torulene and torularhodin	Wild-type: *R. toruloides* AS 2.1389, Mutant: *R. toruloides* HG3	The combination of ultraviolet (UV) radiation and atmospheric and room temperature plasma (ARTP) mutagenesis	• Wild-type: 5.2 mg/g DCW HG3 mutant: 10.5 mg/g DCW	Glucose	[111]

^*^DCW- Dry cell weight, *crtI* (phytoene desaturase), *crtYB (*phytoene synthase*), crtS (*squalene synthase*),crtR (*cytochrome P450 reductase), *crtZ* (β-carotene hydroxylase), *crtW* (β-carotene ketolase), *crtXE* (Carotenoid oxygenase/hydratase), *XYL1* (xylose reductase, *XYL2* (xylitol dehydrogenase), *XKS1* (xylulokinase), *RTO4* (Ras related GTP-binding protein Rho4-like), *RHO3* (Ras homolog family member 3), *RHO4* (Ras homolog family member 4), *RHO5* (Ras homolog family member 5)

## Down-streaming of value-added products

An efficient and reproducible extraction method is essential for accurately evaluating yeast production by oleaginous microbes. Standardizing the extraction procedure is necessary to enable accurate results [16]. Various microorganisms require different extraction methods which vary according to the species. This variation is due to distinct physical properties, such as differing lipid compositions, cell wall structures, and cell shapes [99]. Successful extraction depends on effective disruption of cells of oleaginous microbes. Downstream processing of carotenoids and lipids involves initial biomass separation through centrifugation, filtration, or membrane methods, followed by cell disruption using mechanical (bead milling, homogenization), chemical, or enzymatic techniques. Purification is done using different chromatographic techniques while the last step of concentration is performed using solvent evaporation techniques*.* The membrane processes, such as ultrafiltration and diafiltration, are also used to concentrate and purify lipid or carotenoid extracts [100–102].

### Conventional extraction method for lipids

Different methods have been developed to enhance the recovery of lipids from various species of oleaginous yeast. The major research on lipids in oleaginous yeasts focused on lipid extraction using a modified Folch extraction technique. The separation of lipids in a dual combination of methanol and chloroform is the basic idea of the Folch technique. Hydrogen bonds between proteins and lipids are disrupted by methanol, especially when an organic solvent like chloroform is added. Gravimetric measurements are then used to determine the total fat content. Before extraction of lipid, cell biomass must be pre-treated in order to damage the complex and durable membrane system and cell wall structure. Pre-treatment of such materials is required to speed up the extraction of most intracellular lipids. To release most intracellular lipids for extraction, cells can be broken down using chemical, mechanical, or enzymatic pre-treatment methods [103].

### Conventional extraction method for carotenoids

Researchers have faced the problem of separating various carotenoid compounds that have different polarities.

**Fig. 5:**
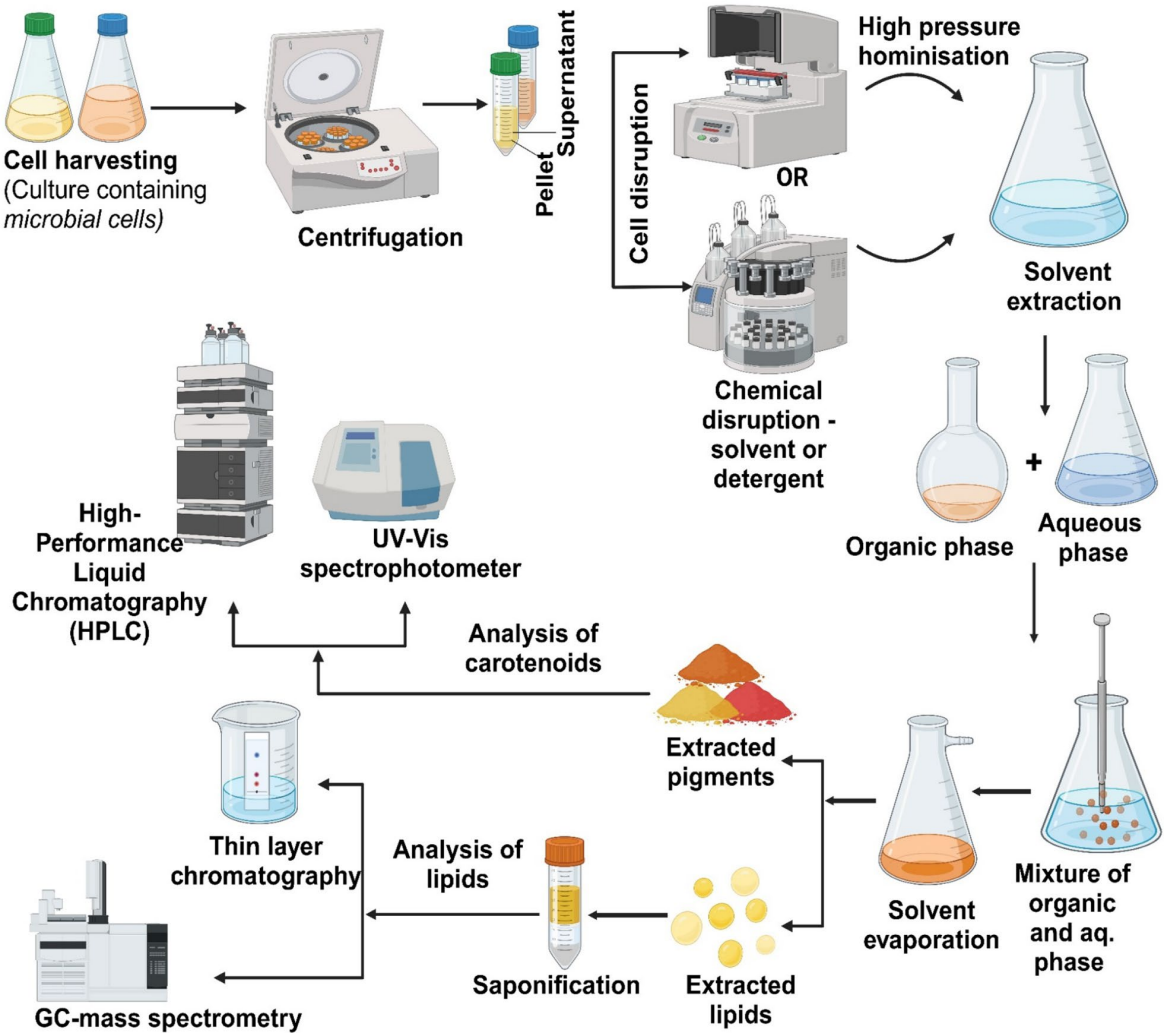
Workflow for extraction and analysis of microbial lipids and carotenoids. The process involves cell harvesting, centrifugation, cell disruption (via high-pressure homogenization or chemical methods), solvent extraction, separation of organic and aqueous phases, and downstream analysis using various techniques

Carotenoids are extracted from the microbial biomass using two steps first, the microbial cell membrane is disrupted, and then the carotenoids are extracted [72]. In order to preserve the quality of carotenoid metabolites, it is essential that carotenoids are handled carefully during the extraction process to avoid deterioration from physical factors like heat, light, and acidity [15]. Conventional extraction procedures for carotenoids require the use of organic solvents due to their hydrophobic nature [100–102]. However, polar solvents such as ethyl acetate, ethanol, or acetone are effective for isolating polar carotenoid molecules. In order to achieve effective extraction, the right solvent must be used, and its polarity must match with the target carotenoid [64, 104]. A study reported that the carotenoids were extracted from *R toruloides* using a modified version of the acetone extraction protocol [64]. In this approach, yeast biomass was first treated with a French press, then extracted using cyclohexane and acetone. During the final stage, acetone was evaporated under a nitrogen atmosphere, leaving behind the carotenoid extract. Saponification of co-extracted lipids is one of the key steps in carotenoid extraction and is often considered essential before analysis [84]. This process removes impurities, such as lipids, that can interfere with the chromatographic detection of carotenoids, thereby improving separation efficiency. The use of methanolic potassium hydroxide for saponification is a standard step in carotenoid analysis (Fig. 5).

### Advanced extraction technologies for lipids and carotenoids

Solvent extraction: The principle behind the solvent extraction technique is"like dissolves like,"according to which polar solvents dissolve polar chemicals (carotenoids) and non-polar solvents preferentially dissolve non-polar compounds (lipids). The target chemicals solubility and polarity influence the solvent selection. Non-polar solvents, such as petroleum ether, hexane, or chloroform, are frequently employed for lipid extraction because they are efficient in solubilizing and extracting non-polar lipids from biomass [105]. On the other hand, polar solvents like acetone, ethanol, or ethyl acetate are recommended for carotenoid extraction because they are capable of efficiently solubilizing and extracting the typically more polar carotenoid molecules [37, 106].

Supercritical fluid extraction (SFE): Supercritical fluids, with properties between liquids and gases, are ideal for extracting non-polar lipids and carotenoids due to their high diffusivity, low viscosity, and adjustable solving power. Due to their inertness, low critical temperature, pressure, and compatibility, supercritical fluids are used for extracting lipids and carotenoids. CO_2_ is the most frequently used supercritical fluid for the extraction process. Both non-polar lipids and carotenoids are selectively extracted from cell biomass using supercritical carbon dioxide, the solvating power may be controlled by temperature, pressure, and co-solvents such ethanol [107, 108].

Enzyme-assisted extraction: This method involves breaking down the components of the cell wall and releasing intracellular substances like carotenoids and lipids. Enzymes such as cellulases, pectinases, or proteases can be employed in lipid extraction procedures to facilitate the non-polar lipids'extraction by dissolving the components of the cell wall, which are proteins, cellulose, or pectin, respectively. The same enzymes can also be used to break down the cell wall and improve the extraction of intracellular carotenoids into the solvent in the case of carotenoid extraction [52].

Ultrasound-assisted extraction: Ultrasonic waves create tiny bubbles in the solvent that burst and generate high pressure and temperature. This helps break the cell wall and allows more metabolites to move into the solvent. In lipid extraction, the non-polar lipids can be more easily moved into the solvent due to the breakdown of the cell wall. In carotenoids extraction, ultrasonic waves'ability to shatter cell walls enhances the release and subsequent extraction of carotenoids into the solvent during the extraction process [52, 109].

Microwave-assisted extraction: In this process cell biomass and solvent mixture is heated up due to molecular friction and dipole rotation caused by microwave radiation. This improves extraction efficiency by helping the solvent to penetrate and break down the cell walls [110, 111, 119].

Saponification: is a step-in carotenoid extraction that removes co-extracted lipids, resulting in a purer extract. This technique converts lipids into fatty acid salts (soaps) and glycerol by hydrolyzing the ester bonds in the lipids with an alkaline solution (such as methanolic KOH). As carotenoids are unaffected by the saponification reaction, this step eliminates the co-extracted lipids and produces a pure carotenoid extract [64, 111] (Fig. 5).

## Beneficial effects of *R. toruloides* on the environment

*R. toruloides* is a potential organism for sustainable biotechnological applications such as waste valorization, wastewater treatment, and bioremediation. This oleaginous yeast is particularly efficient at breaking down harmful substances present in industrial waste streams and converting lignocellulosic biomass into useful metabolites like biofuels and biochemicals. Its unique metabolic capabilities enable it to efficiently utilize both glucose and xylose derived from hydrolyzed lignocellulose, as well as aromatic compounds from lignin, making it an effective agent for biorefinery processes [39]. *R. toruloides* is able to grow in different environment including wastewater, industrial effluents and contaminated water etc. It has the ability to utilize contaminants and producing valuable products like lipids and carotenoids. This makes the effluent more suitable for treatment. Additionally, the modified strains have also demonstrated increased resistance to phenolic inhibitors, which are frequently found in biomass hydrolysates, making it a good option for large-scale uses. *R. toruloides* has shown significant potential in wastewater treatment, providing an economical and environmentally friendly way to reduce pollutants and maintain a sustainable ecosystem. Its ability to reduce the levels of Chemical Oxygen Demand (COD) in industrial effluent is one of its main benefits. For example, research has demonstrated that *R. toruloides* may successfully reduce COD in Palm oil mill effluent (POME) by 43%, demonstrating its capacity to decompose organic waste and promoting environmentally friendly waste disposal methods. This yeast is renowned for its exceptional resistance in wastewater settings that are high in phenol content [112]. By overcoming phenol toxicity and using it as a carbon source, some strains like *R. toruloides 9564 T* can support circular economy and contribute in bioremediation. The biodegradation of phenolic compounds, which are common pollutants in industrial effluents, is essential for reducing environmental risks. Additionally, *R. toruloides* offers a good alternative for inexpensive bioremediation methods, especially in non-sterile wastewater treatment environments [113]. When compared to traditional physicochemical treatment methods, the strain *R. toruloides NCYC 921* has been shown to effectively remove large amounts of contaminants from wastewater without a need for severe sterility conditions. It lowers the operating costs of the process. Hence, *R. toruloides* is a good option for large-scale wastewater treatment applications because of the ability to survive in a variety of environments and reducing pollutants [114]. In addition to its uses in wastewater treatment, *R. toruloides* is essential for the bioremediation of dangerous chemicals, including radioactive and heavy metals. Some *R. toruloides* strains have demonstrated remarkable abilities in the biosorption, bioaccumulation, and bio volatilization of heavy metals, which makes them desirable options for environmentally friendly remediation techniques. *R. toruloides* IR-1395 removes mercury from water by absorbing it, which reduces its bioavailability and lowers the environmental toxicity risks. Due to its high toxicity and stability in aquatic habitats, mercury poisoning poses serious ecological and health risks. *R. toruloides* as a bioremediation agent offers a sustainable substitute for traditional chemical treatments [115]. It has been discovered that the strain *R. toruloides* KS5 can survive and absorb uranium that helps to remove contaminants from polluted areas and restore ecosystems. Uranium pollution, often caused by nuclear and mining activities, poses long-term risks to biodiversity and human health. *R. toruloides* has the potential to be a crucial organism in bioremediation applications aimed at reduce heavy metal and radioactive pollution in contaminated ecosystems because of its metabolic flexibility and capacity to survive in hazardous conditions [116] (Table 5).

**Table 5:** Overview of beneficial characteristics of various *R. toruloides* strains

Strain	Beneficial category	Description	Environmental impact	References
*R. toruloides IR-1395*	Bioremediation of heavy metals, specifically mercury	Remove mercury from aqueous solutions through biosorption, bioaccumulation, and bio volatilization	Potentially cost-effective and eco-friendly method for cleaning up mercury-contaminated water bodies	[115]
*R. toruloides (strain KS5)*	Bioremediation of uranium-contaminated sites	KS5 tolerates up to 6 mM uranium and accumulates 350 mg uranium/g biomass in 48 h	Tolerance and accumulation of high uranium levels in reducing contamination, restoring ecosystems, and minimizing health risks	[116]
*R. toruloides NCYC921*	Waste water treatment	Remove high amounts of pollutants from wastewater at low costs under non-sterile conditions	Enables efficient, cost-effective wastewater treatment and reduces pollution	[127]
*R. toruloides (strain KS5)*	Wastewater treatment	Produces microbial oil and reduces COD of Palm Oil Mill Effluent by 43%	Supports green waste disposal and lowers palm oil production impact	[112]
*R. toruloides 9564 T*	Wastewater treatment and resource recovery	The ability to overcome phenol toxicity at higher concentrations by increasing inoculum size	Treats phenol wastewater and produces valuable lipids, supporting sustainability and circular economy	[113]
*R. toruloides NCYC 921*	Waste valorization	Uses yeast and biorefinery residues to produce biogas via anaerobic digestion	Utilizes waste from yeast production and biorefineries, reducing waste	[128]
*R. toruloides* DSM 4444	Waste valorization and sustainable bioproduction	Use of apple pomace, a waste product from apple processing, as the sole raw material for single cell oil (SCO) production	A byproduct of apple processing, reducing organic waste sent to landfills or needing disposal	[129]
*R. toruloides DSM 4444*	Waste valorization and sustainable bioproduction	Biodiesel-derived glycerol as a sole substrate for non-conventional yeast, converting waste into high-value products and enhancing resource efficiency	A byproduct of biodiesel production, reducing waste and creating a circular economy approach	[130]
*R. toruloides (strain KS5)*	Sustainable production of biodiesel and industrial chemicals	Capable of producing microbial lipids from food processing wastes	Reduces waste by recycling agro-industrial byproducts, contributes to circular economy	[131]
*R. toruloides NCYC 921*	Enzyme production (specifically invertase)	Capable of producing invertase enzyme through fermentation on chitosan-coated magnetic nanoparticles	Magnetic nanoparticles in fermentation enhance enzyme production efficiency, reducing resource use and waste in industrial processes	[132]
*R. toruloides NCYC 921*	Non-conventional oleaginous yeast engineered to produce resveratrol	Sustainable resveratrol production for diverse biological and pharmacological applications	Production of resveratrol offers a more sustainable alternative to plant extraction or chemical synthesis	[133]
*R. toruloides RT1389-3*	Production of ergothioneine, a natural antioxidant with cytoprotective properties	Genetically engineered yeast strain for high-efficiency ergothioneine production	Microbial fermentation for ergothioneine production offers green environmental protection	[134]
*R. toruloides* (genetically engineered with PHB-producing genes)	Sustainable production of biodegradable bioplastics	Non-conventional oleaginous yeast engineered to produce poly-3-D-hydroxybutyrate (PHB),	Offers a bio-based solution with properties comparable to conventional plastics	[135]
*R. toruloides R. toruloides ATCC 10657*	Biodiesel production	Capable of producing microbial oils for biodiesel production using lignocellulosic biomass	Provides a renewable and sustainable alternative to petroleum-based diesel	[115]

Highlighting their roles in environmental remediation, industrial biotechnology, and sustainable resource utilization

## Conclusion

Pigments and lipids produced by *R. toruloides* have shown potential benefits over traditional sources, making them a suitable option for a variety of industrial uses. The food, pharmaceutical, cosmetic, and feed sectors can utilize *R. toruloides*-derived carotenoids, as natural colorants, antioxidants, and nutritional supplements. Similarly, this yeast synthesizes lipids that are useful for production of oleochemicals, nutraceuticals, biodiesel, and palmitic, palmitoleic, stearic, and linoleic acids. Advances in downstream processing methods such as cell disruption, biomass separation, concentration, purification, and formulation have made it possible to recover and use these important microbial products efficiently. *R. toruloides* can utilize diverse carbon sources like lignocellulosic biomass and industrial waste streams. It emerges as a promising microbial factory for sustainable production of carotenoids and lipids. Technological developments in extraction methods have made it easier to extract these important compounds from *R. toruloides* biomass. Metabolic engineering and genetic modification techniques have opened new ways to increase *R. toruloides* yields of lipids and carotenoids. Hence, this microbe gives opportunities be utilized for various industrial applications.

## Data Availability

No datasets were generated or analysed during the current study.

## References

[1] Yadav S, Tiwari K, Gupta C, Tiwari M, Khan A, Sonkar SP. A brief review on natural dyes, pigments: recent advances and future perspectives. Result Chem. 2023;1:47–59.

[2] Barreto J, Casanova L, Junior AN, Reis-Mansur MCPP, Vermelho AB. Microbial pigments: major groups and industrial applications. Microorganisms. 2023;11:300–14. 38138065 10.3390/microorganisms11122920 PMC10745774

[3] Woiciechowski AL, Karp SG, Sobral K, De Carvalho JC, Letti LAJ, Soccol VT, Soccol CR. Pretreatment strategies to enhance value addition of agro-industrial wastes. In: Woiciechowski AL, Karp SG, Sobral K, De Carvalho JC, Letti LAJ, Soccol VT, Soccol CR, editors. Biotransformation of waste biomass into high value biochemicals. Amsterdam: Springer; 2014. p. 29–49.

[4] Chacón-Ordóñez T, Carle R, Schweiggert R. Bioaccessibility of carotenoids from plant and animal foods. J Sci Food Agric. 2019;99:3220–39. 30536912 10.1002/jsfa.9525

[5] Roobha J, Saravanakumar M, Aravindhan K. The effect of light, temperature, pH on stability of anthocyanin pigments in Musa acuminata bract. Res Plant Biol 2011;23:145–55.

[6] Parekh S, Vinci VA, Strobel RJ. Improvement of microbial strains and fermentation processes. Appl Microbiol Biotechnol. 2000;54:287–301. 11030563 10.1007/s002530000403

[7] Sunder S, Gupta A, Kataria R, Ruhal R. Potential of Rhodosporidium toruloides for fatty acids production using lignocellulose biomass. Appl Biochem Biotechnol. 2024;196:2881–900. 37615852 10.1007/s12010-023-04681-w

[8] Kumar A, Vishwakarma HS, Singh J, Dwivedi S, Kumar M. Microbial pigments: production and their applications in various industries. Int J Pharm Chem Biol Sci. 2015;5:203–12.

[9] Passoth V. Lipids of yeasts and filamentous fungi and their importance for biotechnology. In: Passoth V, editor. Biotechnology of yeasts and filamentous fungi. Amsterdam: Springer; 2017. p. 149–204.

[10] Jin M, Slininger PJ, Dien BS, Waghmode S, Moser BR, Orjuela A, Da Costa SL, Balan V. Microbial lipid-based lignocellulosic biorefinery: feasibility and challenges. Trend Biotechnol. 2015;33:469–80. 10.1016/j.tibtech.2014.11.005. 10.1016/j.tibtech.2014.11.005 25483049

[11] Park Y, Nicaud JM, Ledesma-Amaro R. The engineering potential of Rhodosporidium toruloides as a workhorse for biotechnological applications. Trend Biotechnol. 2018;36:739–50. 10.1016/j.tibtech.2017.10.013. 10.1016/j.tibtech.2017.10.013 29132754

[12] Abeln F, Chuck CJ. The history, state of the art and future prospects for oleaginous yeast research. Microb Cell Fact. 2021;20:50. 10.1186/s12934-021-01712-1. 34876155 10.1186/s12934-021-01712-1 PMC8650507

[13] Li Y, Zhao Z. High-density cultivation of oleaginous yeast Rhodosporidium toruloides Y4 in fed-batch culture. Biotechnol Lett. 2007;29:1485–91.

[14] Fei Q, O’Brien M, Nelson R, Chen X, Lowell A, Dowe N. Enhanced lipid production by Rhodosporidium toruloides using different fed-batch feeding strategies with lignocellulosic hydrolysate as the sole carbon source. Biotechnol Bioeng. 2016;113:130–40. 27340432 10.1186/s13068-016-0542-x PMC4918137

[15] Slininger PJ, Dien BS, Quarterman JC, Thompson SR, Kurtzman CP. Screening for oily yeasts able to convert hydrolysates from biomass to biofuels while maintaining industrial process relevance. Method Mol Biol. 2019;1995:249–83. 10.1007/978-1-4939-9484-7_16 31148134

[16] Yu Y, Shi S. Development and perspective of *Rhodotorula toruloides* as an efficient cell factory. J Agric Food Chem. 2023;71:1802–19. 36688927 10.1021/acs.jafc.2c07361

[17] Huang Q, Kamal R, Shen H, Lu H, Song J, Chu Y, Zhao ZK. Pilot-scale conversion of corn stover into lipids by the red yeast *Rhodosporidium toruloides*. J Environ Chem Eng. 2022;10: 106470. 10.1016/j.jece.2021.106470.

[18] Zhao Y, Song B, Li J, Zhang J. *Rhodotorula toruloides*: an ideal microbial cell factory to produce oleochemicals, carotenoids, and other products. World J Microbiol Biotechnol. 2022;38:1–14. 10.1007/S11274-021-03201-4. 10.1007/s11274-021-03201-4 34873661

[19] Kurtzman CP, Fell J, Boekhout T. The yeasts: a taxonomic study. 2nd ed. Amsterdam: Elsevier; 2011.

[20] Zhao X, Hu C, Wu S, Shen H, Zhao ZK. Lipid production by *Rhodosporidium toruloides* Y4 using different substrate feeding strategies. J Ind Microbiol Biotechnol. 2011;38:863–70. 10.1007/s10295-011-0987-6. 20711796 10.1007/s10295-010-0808-4

[21] Scarano P, Naviglio D, Prigioniero A, Tartaglia M, Postiglione A, Sciarrillo R, Guarino C. Sustainability: obtaining natural dyes from waste matrices using the prickly pear peels of *Opuntia ficus-indica* (L.) Miller. Agronomy. 2020;10:226.

[22] Lopes FC, Ligabue-Braun R. Agro-industrial residues: eco-friendly and inexpensive substrates for microbial pigments production. Front Sustain Food Syst. 2021;5: 589414. 10.3389/fsufs.2021.589414.

[23] Rapoport A, Guzhova I, Bernetti L, Buzzini P, Kieliszek M, Kot AM. Carotenoids and some other pigments from fungi and yeasts. Metabolites. 2021;11:92. 10.3390/metabo11020092. 33561985 10.3390/metabo11020092 PMC7915786

[24] Johra FT, Bepari AK, Bristy AT, Reza HM. A mechanistic review of β-carotene, lutein, and zeaxanthin in eye health and disease. Antioxidants. 2020;9:328. 10.3390/antiox9040328. 33114699 10.3390/antiox9111046 PMC7692753

[25] Zafar S, Aqil F. Metal tolerance and biosorption potential of filamentous fungi isolated from metal contaminated agricultural soil. Bioresour Technol. 2007;98:1977–83. 10.1016/j.biortech.2006.09.051. 10.1016/j.biortech.2006.09.051 17113284

[26] Market Data Forecast. Carotenoids market, 2022. https://www.marketdataforecast.com/market-reports/global-carotenoids-market. Accessed 31 Jan 2025.

[27] Honda M, Kageyama H, Hibino T, Ichihashi K, Takada W, Goto M. Isomerization of commercially important carotenoids (lycopene, β-carotene, and astaxanthin) by natural catalysts: isothiocyanates and polysulfides. J Agric Food Chem. 2020;68:3228–37. 32074447 10.1021/acs.jafc.0c00316

[28] Li Z, Li C, Cheng P, Yu G. *Rhodotorula mucilaginosa*—alternative sources of natural carotenoids, lipids, and enzymes for industrial use. Heliyon. 2022;8: e11505. 10.1016/j.heliyon.2022.e11505. 36419653 10.1016/j.heliyon.2022.e11505 PMC9676536

[29] Watkins JL, Pogson BJ. Prospects for carotenoid biofortification targeting retention and catabolism. Trend Plant Sci. 2020;25:1023–32. 10.1016/j.tplants.2020.07.002. 10.1016/j.tplants.2019.12.021 31956035

[30] Burton GW, Mogg TJ, Riley WW, Nickerson JG. β-carotene oxidation products—function and safety. Food Chem Toxicol. 2021;149: 112097. 10.1016/j.fct.2021.112097. 10.1016/j.fct.2021.112207 33891992

[31] Borja-Martínez M, Lozano-Sánchez J, Borrás-Linares I, Pedreño MA, Sabater-Jara AB. Revalorization of broccoli by-products for cosmetic uses using supercritical fluid extraction. Antioxidants. 2020;9:1195. 10.3390/antiox9121195. 33261112 10.3390/antiox9121195 PMC7760773

[32] Mudroňová D, Karaffová V, Koščová J, Bartkovský M, Marcinčáková D, Popelka P. Effect of fungal gamma-linolenic acid and beta-carotene containing prefermented feed on immunity and gut of broiler chicken. Poult Sci. 2018;97:3027–35. 10.3382/ps/pex402. 10.3382/ps/pey306 PMC6305831 30053299

[33] Lee K, Kwon M, Kim Y, Kim Y, Chung MG, Heo SC, Kim Y. β-Carotene regulates cancer stemness in colon cancer in vivo and in vitro. Nutr Res Pract. 2022;16:420–9. 10.4162/nrp.2022.16.4.420. 10.4162/nrp.2022.16.2.161 PMC8971823 35392530

[34] Hambly AJ, Van Duijneveldt JS, Gates PJ. Identification of β-carotene oxidation products produced by bleaching clay using UPLC-ESI-MS/MS. Food Chem. 2021;349: 129261. 10.1016/j.foodchem.2020.129261. 10.1016/j.foodchem.2021.129455 33711704

[35] Barbosa CH, Andrade MA, Séndon R, Silva AS, Ramos F, Vilarinho F, Khwaldia K, Barbosa-Pereira L, Koubaa M. Industrial fruits by-products and their antioxidant profile: can they be exploited for industrial food applications? Foods. 2021;10:272. 10.3390/foods10020272. 33572919 10.3390/foods10020272 PMC7912430

[36] Yang J, Zhang Y, Na X, Zhao A. β-Carotene supplementation and risk of cardiovascular disease: a systematic review and meta-analysis of randomized controlled trials. Nutrients. 2022;14:219. 10.3390/nu14010219. 35334942 10.3390/nu14061284 PMC8950884

[37] Tang Y, Zhang Y, Rosenberg JN, Sharif N, Betenbaugh MJ, Wang F. Efficient lipid extraction and quantification of fatty acids from algal biomass using accelerated solvent extraction (ASE). RSC Adv. 2016;6:20811–8. 10.1039/c6ra02747a.

[38] Liu Z, Radi M, Mohamed E, Feist AM, Dragone G, Mussatto SI. Adaptive laboratory evolution of Rhodosporidium toruloides to inhibitors derived from lignocellulosic biomass and genetic variations behind evolution. Bioresour Technol. 2021;322: 124470. 10.1016/j.biortech.2021.124470. 33894448 10.1016/j.biortech.2021.125171

[39] Yaegashi J, Kirby J, Ito M, Sun J, Dutta T, Mirsiaghi M, Sundstrom ER, Rodriguez A. Rhodosporidium toruloides: a new platform organism for conversion of lignocellulose into terpene biofuels and bioproducts. Biotechnol Biofuel. 2017;10:241. 10.1186/s13068-017-0851-0. 10.1186/s13068-017-0927-5 PMC5651578 29075325

[40] Kot AM, Błażejak S, Kurcz A, Gientka I, Kieliszek M. *Rhodotorula glutinis*—potential source of lipids, carotenoids, and enzymes for use in industries. Appl Microbiol Biotechnol. 2016;100:6103–17. 10.1007/s00253-016-7572-0. 27209039 10.1007/s00253-016-7611-8 PMC4916194

[41] Zhang C, Shen H, Zhang X, Yu X, Wang H, Xiao S, Wang J, Zhao ZK. Combined mutagenesis of *Rhodosporidium toruloides* for improved production of carotenoids and lipids. Biotechnol Lett. 2016;38:1733–8. 10.1007/s10529-016-2187-2. 27311308 10.1007/s10529-016-2148-6

[42] Chattopadhyay A. Lipid production by oleaginous yeasts. In: Chattopadhyay A, editor. Microbiol Appl. Amsterdam: Elsevier; 2021. 10.1016/bs.aambs.2021.03.003 34353502

[43] Poontawee R, Lorliam W, Polburee P. Oleaginous yeasts: biodiversity and cultivation. Front Sustain Biotechnol. 2023;44:100295.

[44] Ji X, Huang H. Engineering microbes to produce polyunsaturated fatty acids. Trend Biotechnol. 2019;37:1250–62. 10.1016/j.tibtech.2019.04.007. 10.1016/j.tibtech.2018.10.002 30376959

[45] Vasconcelos B, Teixeira JC, Dragone G, Teixeira JA. Oleaginous yeasts for sustainable lipid production—from biodiesel to surf boards, a wide range of “green” applications. Appl Microbiol Biotechnol. 2019;103:3651–67. 10.1007/s00253-019-09880-x. 30911785 10.1007/s00253-019-09742-x

[46] Lakshminarayana R, Baskaran V. Influence of olive oil on the bioavailability of carotenoids. Eur J Lipid Sci Technol. 2013;115:1085–93.

[47] Kot A, Błażejak S, Gientka I, Kieliszek M. Torulene and torularhodin: “new” fungal carotenoids for industry? Microb Cell Fact. 2018;17:49. 29587755 10.1186/s12934-018-0893-z PMC5870927

[48] Zimmer T, Mendonça C, Zambiazi RC. Methods of protection and application of carotenoids in foods—a bibliographic review. Food Biosci. 2022;48:101829.

[49] Shen H, Zhang X, Gong Z, Wang Y, Yu X, Yang X, Zhao ZK. Compositional profiles of *Rhodosporidium toruloides* cells under nutrient limitation. Appl Microbiol Biotechnol. 2017;101:3801–9. 28168317 10.1007/s00253-017-8157-0

[50] Huang X, Liu J, Lu L, Peng K. Culture strategies for lipid production using acetic acid as sole carbon source by *Rhodosporidium toruloides*. Biotechnol Adv. 2016;206:141–9. 10.1016/j.biortech.2016.01.073 26851898

[51] Zhang Y, Peng J, Zhao H. Engineering oleaginous yeast *Rhodotorula toruloides* for overproduction of fatty acid ethyl esters. Biotechnol Biofuel. 2021;14:115. 10.1186/s13068-021-01965-3 PMC8106135 33964988

[52] Saini R, Hegde K, Osorio-Gonzalez CS, Brar SK, Vezina P. Evaluating the potential of *Rhodosporidium toruloides*-1588 for high lipid production using undetoxified wood hydrolysate as a carbon source. Energies. 2020;13:5960.

[53] Chen HY, Lei JY, Li SL, Guo LQ, Lin JF, Wu GH, Lu J, Ye ZW. Progress in biological activities and biosynthesis of edible fungi terpenoids. Crit Rev Food Sci Nutr. 2023;63:7288–310. 35238261 10.1080/10408398.2022.2045559

[54] Vranová E, Coman D, Gruissem W. Network analysis of the MVA and MEP pathways for isoprenoid synthesis. Annu Rev Plant Biol. 2013;64:665–700. 23451776 10.1146/annurev-arplant-050312-120116

[55] Zhuang X, Kilian O, Monroe E, et al. Monoterpene production by the carotenogenic yeast *Rhodosporidium toruloides*. Microb Cell Fact. 2019. 10.1186/s12934-019-1099-8. 30885220 10.1186/s12934-019-1099-8 PMC6421710

[56] Miziorko HM. Enzymes of the mevalonate pathway of isoprenoid biosynthesis. Arch Biochem Biophys. 2011;505:150–6. 10.1016/j.abb.2010.09.028 PMC3026612 20932952

[57] Pham KD, Hakozaki Y, Takamizawa T, Yamazaki A, Yamazaki H, Mori K, Aburatani S. Analysis of the light regulatory mechanism in carotenoid production in *Rhodosporidium toruloides* NBRC 10032. Biosci Biotechnol Biochem. 2021;85:399–408. 10.1093/bbb/zbab109 34124766

[58] Fang N, Wang C, Liu X, Zhao X, Liu Y. De novo synthesis of astaxanthin: from organisms to genes. Biotechnol Adv. 2019. 10.1016/j.tifs.2019.08.016.31626951

[59] Chatzivasileiou AO, Ward V, McBride ES, Stephanopoulos G. Two-step pathway for isoprenoid synthesis. Proc Natl Acad Sci U S A. 2019;116:506–11. 30584096 10.1073/pnas.1812935116 PMC6329939

[60] Ma T, Deng Z, Liu T. Microbial production strategies and applications of lycopene and other terpenoids. World J Microbiol Biotechnol. 2016;32:1–9. 26715120 10.1007/s11274-015-1975-2

[61] Wang L, Liu Z, Jiang H, Mao X. Biotechnology advances in β-carotene production by microorganisms. Trend Food Sci Technol. 2021;115:1085–93.

[62] Hernández-Almanza A, Montanez J, Martínez G, Aguilar-Jiménez A, Contreras-Esquivel JC. Lycopene: progress in microbial production. Trends Food Sci Technol. 2016;56:88–97.

[63] Beopoulos A, Nicaud JM, Gaillardin C. An overview of lipid metabolism in yeasts and its impact on biotechnological processes. Appl Microbiol Biotechnol. 2011;90:1193–206. 21452033 10.1007/s00253-011-3212-8

[64] Reif C, Arrigoni E, Schärer H, et al. Carotenoid database of commonly eaten Swiss vegetables and their estimated contribution to carotenoid intake. J Food Compos Anal. 2013;32:1–9.

[65] Tkachenko AF, Tigunova OA, Shulga SM. Microbial lipids as a source of biofuel. Cytol Genet. 2013;47:343–8. 24437195

[66] Kennedy EP. Biosynthesis of complex lipids. Biochem Biophys Res Commun. 1961;47:345–8.

[67] Gong G, Zhang X, Liu Q. Simultaneously enhanced intracellular lipogenesis and β-carotene biosynthesis of *Rhodotorula glutinis* by light exposure with sodium acetate as the substrate. Appl Microbiol Biotechnol. 2020;295:122274. 10.1016/j.biortech.2019.122274 31670113

[68] Xie ZT, Mi BQ, Lu YJ, Chen MT, Ye ZW. Research progress on carotenoid production by *Rhodosporidium toruloides*. Appl Microbiol Biotechnol. 2024;108:1–14. 38170311 10.1007/s00253-023-12943-0 PMC13497458

[69] Sun W, Yang X, Wang X, Lin X, Wang Y, Zhang S, Luan Y, Zhao ZK. Homologous gene targeting of a carotenoids biosynthetic gene in *Rhodosporidium toruloides* by agrobacterium-mediated transformation. Biotechnol Lett. 2017;39:1001–7. 28337556 10.1007/s10529-017-2324-3

[70] Sinha S, Singh G, Paul D. Lipid and carotenoid production by *Rhodosporodium toruloides* ATCC 204091 using C5 and C6 sugars obtained from lignocellulosic hydrolysate. J Environ Biol. 2021;42:938–44.

[71] Cardoso L, Karp S, Vendruscolo F, Zoz KY, Carvalho JC. Biotechnological production of carotenoids and their applications in food and pharmaceutical products. Carotenoids. 2017. 10.5772/67725.

[72] Castaldo L, Trombetti S, Papapostolou H, Kachrimanidou V, Alexandri M, Plessas S, Papadaki A, Kopsahelis N. Natural carotenoids: recent advances on separation from microbial biomass and methods of analysis. Antioxidants. 2023;12:1030. 10.3390/antiox12051030. 37237896 10.3390/antiox12051030 PMC10215962

[73] Shaigani P, Awad D, Redai V, Fuchs M, Haack M, Mehlmer N, Brueck T. Oleaginous yeasts—substrate preference and lipid productivity: a view on the performance of microbial lipid producers. Microb Cell Fact. 2021;20:50. 34876116 10.1186/s12934-021-01710-3 PMC8650408

[74] Ammar EM, Arora N, Philippidis GP. The prospects of agricultural and food residue hydrolysates for sustainable production of algal products. Energies. 2020;13:6427.

[75] Carvalho AKF, Bento HBS, Reis CER, Castro HF. Sustainable enzymatic approaches in a fungal lipid biorefinery based in sugarcane bagasse hydrolysate as carbon source. Bioresour Technol. 2019;288: 121634. 10.1016/j.biortech.2018.12.118 30640021

[76] de Almeida SGC, Souza JP, Fogarin HM, Franca BV, Dussán KJ. Assessment of lipid synthesis from sugarcane biomass by adaptive strains of *Rhodosporidium toruloides*. Biomass Conv Biorefin. 2024. 10.1007/S13399-024-05499-0.

[77] Gupta PL, Choi H-J, Pawar RR, Jung SP, Lee S-M. Enhanced biomass production through optimization of carbon source and utilization of wastewater as a nutrient source. J Environ Manag. 2016;183:185–93. 10.1016/j.jenvman.2016.10.018. 10.1016/j.jenvman.2016.10.018 27789093

[78] Takano H, Obitsu S, Beppu T, Ueda K. Light-induced carotenogenesis in *Streptomyces coelicolor* A3(2): Identification of an extracytoplasmic function sigma factor that directs photodependent transcription of the carotenoid biosynthesis gene cluster. J Bacteriol. 2005;187:1825–32. 15716454 10.1128/JB.187.5.1825-1832.2005 PMC1064024

[79] Khanafari A, Tayari K, Emami M. 2008. Light requirement for the carotenoids production by Mucor hiemalis. Iranian Journal of Basic Medical Sciences: Mashhad.

[80] Pham KD, Shida Y, Miyata A, et al. Effect of light on carotenoid and lipid production in the oleaginous yeast *Rhodosporidium toruloides*. Biosci Biotechnol Biochem. 2020;84:1501–12. 32189572 10.1080/09168451.2020.1740581

[81] Pinheiro MJ, Bonturi N, Belouah I, et al. Xylose metabolism and the effect of oxidative stress on lipid and carotenoid production in *Rhodotorula toruloides*: insights for future biorefinery. Front Bioeng Biotechnol. 2020. 10.3389/FBIOE.2020.01008/FULL. 32974324 10.3389/fbioe.2020.01008 PMC7466555

[82] Bertacchi S, Cantù C, Porro D, Branduardi P. Optimization of carotenoids production from *Camelina sativa* meal hydrolysate by *Rhodosporidium toruloides*. Fermentation. 2021;7:208. 10.3390/fermentation7040208.

[83] Wang Q, Guo F, Rong Y. Lipid production from hydrolysate of cassava starch by *Rhodosporidium toruloides* 21167 for biodiesel making. Renew Energ. 2012;43:15–20.

[84] Qi F, Shen P, Hu R, Xue T, Jiang X, Qin L, Chen Y, Huang J. Carotenoids and lipid production from *Rhodosporidium toruloides* cultured in tea waste hydrolysate. Biotechnol Biofuels. 2020;13:145. 10.1186/s13068-020-01712-0. 32322304 10.1186/s13068-020-01712-0 PMC7161300

[85] Zhang S, Ito M, Skerker JM, Arkin AP, Rao CV. Metabolic engineering of the oleaginous yeast *Rhodosporidium toruloides* IFO0880 for lipid overproduction during high-density fermentation. Appl Microbiol Biotechnol. 2016;100:9393–405. 27678117 10.1007/s00253-016-7815-y

[86] Fillet S, Ronchel C, Callejo C, et al. Engineering *Rhodosporidium toruloides* for the production of very long-chain monounsaturated fatty acid-rich oils. Appl Microbiol Biotechnol. 2017;101:7271–80. 28812146 10.1007/s00253-017-8461-8

[87] Sankari M, Hridya H, Sneha P, et al. Implication of salt stress induces changes in pigment production, antioxidant enzyme activity, and qRT-PCR expression of genes involved in the biosynthetic pathway of Bixa orellana L. Funct Integr Genomics. 2019;19:565–74. 30694406 10.1007/s10142-019-00654-7

[88] Li C, Zhang N, Li B, et al. Increased torulene accumulation in red yeast *Sporidiobolus pararoseus* NGR as stress response to high salt conditions. Food Chem. 2017;237:1041–7. 28763948 10.1016/j.foodchem.2017.06.033

[89] Illarionov A, Lahtvee PJ, Kumar R. Potassium and sodium salt stress characterization in the yeasts *Saccharomyces cerevisiae*, *Kluyveromyces marxianus*, and *Rhodotorula toruloides*. Appl Environ Microbiol. 2021;87:1–16. 10.1128/AEM.03100-20 PMC8315938 33893111

[90] Singh G, Sinha S, Bandyopadhyay KK, Lawrence M, Paul D. Triauxic growth of an oleaginous red yeast *Rhodosporidium toruloides* on waste “extract” for enhanced and concomitant lipid and β-carotene production. Microb Cell Fact. 2018;17:182. 30454058 10.1186/s12934-018-1026-4 PMC6240951

[91] Tchakouteu SS, Kopsahelis N, Chatzifragkou A, et al. *Rhodosporidium toruloides* cultivated in NaCl-enriched glucose-based media: adaptation dynamics and lipid production. Eng Life Sci. 2017;17:237–48. 32624771 10.1002/elsc.201500125 PMC6999334

[92] Wu CC, Honda K, Kazuhito F. Current advances in alteration of fatty acid profile in Rhodotorula toruloides: a mini-review. World J Microbiol Biotechnol. 2023;39:234. 37358633 10.1007/s11274-023-03595-3 PMC10293357

[93] Igreja WS, Maia FA, Lopes AS, Chisté RC. Biotechnological production of carotenoids using low cost-substrates is influenced by cultivation parameters: a review. Int J Mol Sci. 2021;22: 168819. 10.3390/ijms22168819. 10.3390/ijms22168819 PMC8396175 34445525

[94] Kamal R, Liu Y, Li Q, et al. Exogenous l-proline improved *Rhodosporidium toruloides* lipid production on crude glycerol. Biotechnol Biofuels. 2020;13:177. 10.1186/s13068-020-01798-6. 32944075 10.1186/s13068-020-01798-6 PMC7490893

[95] Zhu Z, Zhang S, Liu H, et al. A multi-omic map of the lipid-producing yeast *Rhodosporidium toruloides*. Nat Commun. 2012;3:1058. 10.1038/ncomms2112. 23047670 10.1038/ncomms2112 PMC3493640

[96] Xu J, Liu D. Exploitation of genus *Rhodosporidium* for microbial lipid production. World J Microbiol Biotechnol. 2017;33:15. 28220353 10.1007/s11274-017-2225-6

[97] Zhang S, Skerker JM, Rutter CD, et al. Engineering *Rhodosporidium toruloides* for increased lipid production. Biotechnol Bioeng. 2016;113:1056–66. 26479039 10.1002/bit.25864

[98] Bonturi N, Crucello A, Viana A, et al. Microbial oil production in sugarcane bagasse hemicellulosic hydrolysate without nutrient supplementation by a *Rhodosporidium toruloides* adapted strain. Bioresour Technol. 2017;57:16–25.

[99] Kim J, Lee EJ, Lee KE, et al. Lipid extract derived from newly isolated *Rhodotorula toruloides* LAB-07 for cosmetic applications. Comput Struct Biotechnol J. 2023;21:327–38. 10.1016/j.csbj.2023.03.018 PMC10036517 36968014

[100] Sereti F, Alexandri M, Papadaki A, Papapostolou H, Kopsahelis N. Natural lycopene and β-carotene synthesis by *Rhodosporidium kratochvilovae* yeasts: sustainable production, chemical characterization and antioxidative properties. Food Biosci. 2024;47:10412.

[101] Karamerou E, Wensel B. Cultivation modes for microbial oil production using oleaginous yeasts–a review. Biochem Engin J. 2019. 10.1016/j.bej.2019.107322.

[102] Ibrahim S, Wei Lie Chin GJ, Misson M. Enhanced microbial biomass and lipid production through co-cultivation of yeast *Rhodotorula toruloides* and microalga *Chaetoceros muelleri*. Malays J Microbiol. 2022;23:167–74.

[103] JingYang XX, XueBing ZZ, Du WD. Bioconversion of glycerol into lipids by *Rhodosporidium toruloides* in a two-stage process and characterization of lipid properties. Eng Life Sci. 2017. 10.1002/elsc.201600062. 10.1002/elsc.201600062 PMC6999356 32624776

[104] Nagaraj Y, Burkina V, Okmane L, Fermentation JB. Identification, quantification, and kinetic study of carotenoids and lipids in *Rhodotorula toruloides* CBS 14 cultivated on wheat straw hydrolysate. Fermentation. 2022;8(7):300. 10.3390/fermentation8070300.

[105] Villegas-Méndez M, Montañez J, Contreras-Esquivel JC, Salmerón I, Koutinas AA. Scale-up and fed-batch cultivation strategy for the enhanced co-production of microbial lipids and carotenoids using renewable waste feedstock. J Environ Manag. 2023;319: 115752. 10.1016/j.jenvman.2023.117866 37030236

[106] Bourdon L, Jensen AA, Kavanagh JM. Microalgal production of zeaxanthin. Algal Res. 2021;56: 102278.

[107] Fernández-Acosta K, Morais J, Balcázar C, Ortega M, Jiménez A. Evaluation of different variables on the supercritical CO2 extraction of oat (*Avena sativa* L.) oil; main fatty acids, polyphenols, and antioxidant content. J Cereal Sci. 2019;88: 102776.

[108] Solana M, Rizza C, Della PG. Exploiting microalgae as a source of essential fatty acids by supercritical fluid extraction of lipids: comparison between *Scenedesmus obliquus*. Chlorella Algal Res. 2014;6:8–15.

[109] Dos Reis AS, Santos AS, De Carvalho Gonçalves JF, Silva A, Reis D. Ultrasound-assisted lipid extractions, enriched with sterols and tetranortriterpenoids, from *Carapa guianensis* seeds and the application of lipidomics using GC/MS. RSC Adv. 2021;11(29):17694–705. 10.1039/d1ra04776k. 35493601 10.1039/d1ra04776k PMC9042240

[110] Zhou X, Jin W, Tu R, Guo Q, Han S, Chen C, Wang Q, Liu W. Optimization of microwave-assisted lipid extraction from microalga *Scenedesmus obliquus* grown on municipal wastewater. J Clean Prod. 2019;238: 117845.

[111] Ji C, Li S, Sun Z, Lv J, Liang H, Yang Z, Xu W, Zhang S, Lin X. Analysis of carotenoid profile changes and carotenogenic genes transcript levels in *Rhodosporidium toruloides *mutants from an optimized agrobacterium tumefaciens. Biotechnol Appl Biochem. 2021;68:71–81. 32017256 10.1002/bab.1895

[112] Justine I, Chin GJWL, Yong WT, Misson M. Cultivation of rhodotorula toruloides using palm oil mill effluent: effect on the growth, lipid production, and waste removal. Pertanika J Sci Technol. 2022;30:2477–91.

[113] Singh S, Bharadwaj T, Verma D, Dutta K. Valorization of phenol contaminated wastewater for lipid production by *Rhodosporidium toruloides* 9564T. Chemosphere. 2022;286: 131566. 36057352 10.1016/j.chemosphere.2022.136269

[114] Schultz JC, Mishra S, Gaither E, Mejia A, Dinh H, Maranas C, Zhao H. Metabolic engineering of *Rhodotorula toruloides* IFO0880 improves C16 and C18 fatty alcohol production from synthetic media. Microb Cell Fact. 2022;21:140. 10.1186/s12934-022-01750-3. 35183175 10.1186/s12934-022-01750-3 PMC8858515

[115] Patel A, Arora N, Sartaj KM, Pruthi V, Pruthi PA. Sustainable biodiesel production from oleaginous yeasts utilizing hydrolysates of various non-edible lignocellulosic biomasses. Renew Sustain Energ Rev. 2016;64:100–16.

[116] Gerber U, Huebner R, Rossberg A, Krawczyk-Baersch E, Merroun ML. Metabolism-dependent bioaccumulation of uranium by *Rhodosporidium toruloides* isolated from the flooding water of a former uranium mine. PLoS ONE. 2018;13(11): e0201903. 10.1371/journal.pone.0201903. 30089169 10.1371/journal.pone.0201903 PMC6082562

[117] Bertacchi S, Cantù C, Porro D, Branduardi P. Optimization of carotenoids production from camelina sativa meal hydrolysate by Rhodosporidium toruloides. Fermentation. 2021;7(4):208. 10.3390/fermentation7040208.

[118] Pereira RN, Silveira JM, Burkert JF, Ores JC, Burkert CAV. Simultaneous lipid and carotenoid production by stepwise fed-batch cultivation of *Rhodotorula Mucilaginosa* with crude glycerol. Braz J Chem Eng. 2019;36(3):1099–108.

[119] Fallahi S, Habibi A, Abbasi S, Sharifi R. Optimized fed-batch cultivation of *Rhodotorula toruloides* in a bubble column bioreactor progressed the β-carotene production from corn steep liquor. Braz J Microbiol. 2023;54:2719–31. 37783938 10.1007/s42770-023-01137-5 PMC10689328

[120] Martins V, Dias C, Caldeira J, Duarte L, Rosa AR. Carob pulp syrup: a potential mediterranean carbon source for carotenoids production by *Rhodosporidium toruloides* NCYC 921. Algal Res. 2018;35:232–9.

[121] Martins LC, Palma M, Angelov A, Nevoigt E, Liebl W, Sá-Correia I. Complete utilization of the major carbon sources present in sugar beet pulp hydrolysates by the oleaginous red yeasts *Rhodotorula toruloides* and *R. mucilaginosa*. J Fungi. 2021;7(10):833. 10.3390/jof7100833. 10.3390/jof7030215 PMC8002571 33802726

[122] Xiangfeng HH, Jianan LL, Lijun LL. Culture strategies for lipid production using acetic acid as sole carbon source by *Rhodosporidium toruloides*. Biotechnol Lett. 2016;38(2):239–45. 10.1016/j.biortech.2016.01.073 26851898

[123] Yang X, Sun W, Shen H, Zhang S, Jiao X. Expression of phosphotransacetylase in *Rhodosporidium toruloides* leading to improved cell growth and lipid production. RSC Adv. 2018;8:22515–23. 10.1039/c8ra03028f. 35539198 10.1039/c8ra03028f PMC9082159

[124] Ye Z, Sun T, Hao H, He Y, Liu X, Guo M. Optimising nutrients in the culture medium of *Rhodosporidium toruloides* enhances lipid production. AMB Express. 2021;11:149. 10.1186/s13568-021-01258-2. 34778908 10.1186/s13568-021-01313-6 PMC8590987

[125] Machado WRM, Silva LG, Vanzela ESL, Del Bianchi VL. Production of carotenoids by *Rhodosporidium toruloides* isolated from Brazilian tropical savannah. Int Food Res J. 2019;26:1259–67.

[126] Lin X, Gao N, Liu S, Zhang S, Song S, Ji C, Dong X, Su Y, Zhao ZK, Zhu B. Characterization of carotenoid productions and profiles of three *Rhodosporidium toruloides* mutants from *Agrobacterium *tumefaciens-mediated transformation. Biotechnol Lett. 2017;34:335–42. 10.1002/yea.3236 28426167

[127] Mohiuddin O, Harvey A, Ledesma MTO, Velasquez-Orta S. Bioremediation of waste by yeast strains. Electron J Biotechnol. 2024;59:29–35.

[128] Batista AP, López EP, Dias C, da Silva TL, Marques IP. Wastes valorization from *Rhodosporidium toruloides* NCYC 921 production and biorefinery by anaerobic digestion. Bioresour Technol. 2017;225:56–64. 10.1016/j.biortech.2016.10.042. 10.1016/j.biortech.2016.11.113 27992793

[129] Tuhanioglu A, Hamamci H, Alpas H, Cekmecelioglu D. Valorization of apple pomace via single-cell oil production using oleaginous yeast *Rhodosporidium toruloides*. Wast Biomass Valoriz. 2023;14:765–79.

[130] Biotechnological valorization of biodiesel-derived glycerol. Trials with the non-conventional yeasts Yarrowia lipolytica and Rhodosporidium sp. Biotechnol Lett. 2020;42:2211–7.32488441

[131] Donzella S, Serra I, Fumagalli A, Pellegrino L, Mosconi G, Lo SR, Compagno C. Recycling industrial food wastes for lipid production by oleaginous yeasts *Rhodosporidiobolus azoricus* and *Cutaneotrichosporon oleaginosum*. Biotechnol Biofuels Bioprod. 2022;15:107. 10.1186/s13068-022-02149-3. 35568880 10.1186/s13068-022-02149-3 PMC9107756

[132] Alonso-Estrada D, et al. Invertase production by *Rhodotorula toruloides* in submerged and surface adhesion on magnetic nanoparticles fermentations. Biotechnol. 2024;65:134–45.

[133] Zhang M, Gao Q, Liu Y, Fang Z, Gong Z, Zhao ZK, Yang X. Metabolic engineering of *Rhodosporidium toruloides* for resveratrol production. Microb Cell Fact. 2022;21:270. 36566171 10.1186/s12934-022-02006-w PMC9789595

[134] Li L, Xu S, Jiang Y. Research progress in the production of ergothioneine by biosynthesis. Biotech Bull. 2024;5:12–9.

[135] Lindh T, Brink D, Gorwa-Grauslund MF, Carlquist M. Engineering *Rhodosporidium toruloides* for the production of polyhydroxybutyrate. Appl Microbiol Biotechnol. 2021;105:1211–20.

[136] Bertacchi S, Bettiga M, Porro D, Branduardi P. *Camelina sativa* meal hydrolysate as sustainable biomass for the production of carotenoids by *Rhodosporidium toruloides*. Biotechnol Biofuels. 2020;13:1–10. 32190112 10.1186/s13068-020-01682-3 PMC7066749

